# Casein kinase I isoforms contribute to platelet activation and thrombogenesis via RIPK3–MLKL signaling

**DOI:** 10.1038/s42003-025-08868-1

**Published:** 2025-09-30

**Authors:** Vipin Singh, Mohammad Ekhlak, Susheel N. Chaurasia, Debabrata Dash

**Affiliations:** Centre for Advanced Research on Platelet Signaling and Thrombosis Biology, Department of Biochemistry, Institute of Medical Sciences, Banaras Hindu University, Varanasi, India

**Keywords:** Medical research, Cell signalling

## Abstract

Platelets are small, enucleate blood cells having life span of 10-12 days that play fundamental role in hemostasis and thrombosis. Casein Kinase 1 (CK1) is a serine/threonine-specific protein kinase that governs multiple cellular processes including circadian rhythm, morphogen signaling and apoptosis; however, its role in platelet biology and thrombogenesis remains unexplored. Employing a CK1-specific pharmacological inhibitors, we demonstrate here a pivotal role of CK1 in agonist-induced platelet activation. Inhibition of CK1 disrupts platelet functions that include aggregation, integrin activation, interaction with leukocytes, and thrombus formation under arterial shear ex vivo as well as in a murine model of thrombosis. CK1 maintains mitochondrial integrity by stabilizing inner mitochondrial membrane that propels energy metabolism in activated platelets. Notably, CK1 inhibition suppresses phosphorylation of receptor-interacting protein kinase 3 (RIPK3) and mixed lineage kinase domain-like protein (MLKL), key arbiters of platelet activation leading to necroptosis, thus mechanistically linking CK1 activity to platelet prothrombotic responses. Downregulation of CK1 did not affect primary hemostasis nor platelet viability while significantly deferring thrombus formation, which underscores its potential as a safe therapeutic option against thrombotic disorders. This study uncovers an emerging role of CK1 in unleashing of prothrombotic phenotype and positions CK1 as a potential target for antithrombotic measures.

## Abstract (web summary)

CK1 drives platelet activation and thrombosis via RIPK3–MLKL signaling, sustaining mitochondrial integrity and energy metabolism. Its inhibition curbs thrombosis without affecting viability or primary hemostasis, offering a safe antithrombotic target.

## Introduction

Enucleate platelets, the principal hemostatic agents in blood, circulate in non-adhesive quiescent states. Platelets become activated at sites of vascular damage and adhere to exposed sub-endothelial matrix components^1^. In this process, they change their morphology from discoid to spread state and join with neighboring platelets via fibrinogen bridging to form hemostatic plugs. This response is termed ‘hemostasis’^2^. A similar but more exaggerated response from platelets upon rupture of an atherosclerotic plaque leads to arterial thrombosis with potentially fatal consequences, such as myocardial infarction and ischemic stroke^3^. Comprehension of platelet activity is, therefore, imperative for understanding the mechanistic underpinning of thrombotic disorders and developing therapeutic strategies.

Casein kinase-1 (CK1) is a family of serine/threonine protein kinases comprising seven human isoforms, namely α, γ1, γ2, γ3, δ, ε and less described α-like^4^, of which α, δ and ε are the best studied till date. CK1 remains conserved from yeast to human, which underscores its fundamental role in cellular homeostasis and biological processes^5^. CK1 isoforms have a highly conserved amino-terminal kinase domain that targets a variety of substrates^6,7^. CK1 governs a wide repertoire of functions ranging from regulation of circadian rhythm to DNA repair, cell proliferation, inflammation, apoptosis, as well as membrane trafficking^8–10^. CK1 is a potent modulator of cellular circadian rhythm as it phosphorylates PER1, a clock protein, leading to its degradation^11^ and is thus associated with sleep disturbances^12^. It plays a vital role in morphogen signaling through phosphorylation of β-catenin^13^ and Gli3^14^, which are the effector mediators of the Wnt and Hedgehog pathways, respectively. In a compelling investigation, Hanna-Addams and colleagues have demonstrated the pivotal role of CK1 in phosphorylation of receptor-interacting protein kinase 3 (RIPK3) and mixed lineage kinase domain-like (MLKL), the key drivers of necroptosis^15^. Dysregulation of CK1 activity has been implicated in multiple diseases such as cancer, neurodegenerative disorders and cardiovascular ailments, highlighting its potential as a promising therapeutic target^6,16,17^.

Several signaling pathways in which CK1 plays a significant role are known to be operational in platelets. These include Wnt^18,19^ and Sonic hedgehog^20^ morphogen pathways as well as necroptosis signaling^21^. While CK1’s presence in human platelets has long been recognized^19^, its contribution to platelet biology remains uncharted. Employing a pharmacological inhibitor, we demonstrate in this study a critical role of CK1 in the transformation of agonist-challenged platelets to hemostatically active ‘prothrombotic’ units, potentially driven by CK1-mediated phosphorylation of RIPK3 and MLKL. CK1 exerts a stabilizing effect on the platelet inner mitochondrial membrane, impacting energy metabolism, which, thus, projects the enzyme as a potential therapeutic target against thrombotic cardiovascular episodes.

## Results

### Casein kinase-1 activity boosts platelet hemostatic responses to physiological agonists

**Fig. 1:**
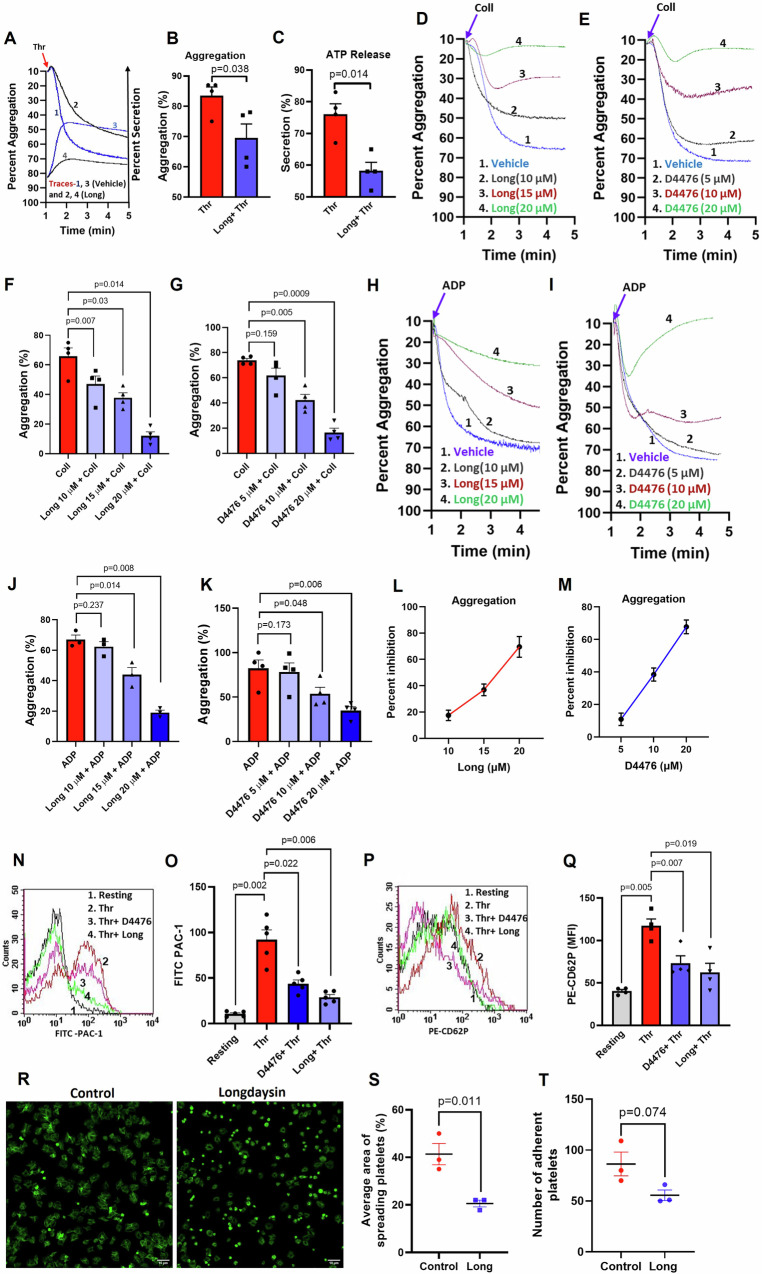
Casein kinase-1 governs agonist-induced platelet activation responses. **A** Tracing representing aggregation of washed human platelets induced by thrombin (0.25 U/ml) in the absence (tracing 1) or presence of longdaysin (15 µM) (tracing 2). respectively. Tracings 3 and 4, secretion of adenine nucleotides from platelet dense granules induced by thrombin in the presence of vehicle or longdaysin, respectively. **B**, **C** Corresponding bar diagrams denoting mean platelet aggregation and secretion of adenine nucleotides, respectively. **D**, **E** Tracings representing aggregation of washed human platelets induced by collagen (2 µg/ml) in the absence or presence of longdaysin (**D**) and D4476 (**E**), as indicated. **F**, **G** Corresponding bar picture representing mean platelet aggregation. **H**, **I** Platelet aggregation in PRP induced by ADP (5 µM) in the absence and presence of longdaysin (**H**) and D4476 (**I**), as indicated. **J**, **K** Corresponding bar diagrams showing mean platelet aggregation. **L**, **M** Curves showing dose-dependent inhibition of either collagen- or ADP-induced platelet aggregation by longdaysin and D4476, respectively. **N**, **P** Histograms representing binding of FITC-PAC-1 and PE-anti-CD62P, respectively, to platelets pre-treated with thrombin in the presence or absence of longdaysin and D4476, as specified. **O**, **Q** Corresponding bar diagrams representing mean fluorescence intensity. **R** Confocal microscopy of platelet adhesion and spreading on immobilized fibrinogen. Washed platelets pre-added with vehicle or longdaysin were allowed to spread on immobilized fibrinogen for 20 min at 37 °C, followed by staining with FITC-conjugated phalloidin. **S**, **T** Corresponding scatter dot plots denoting the number of adhering platelets and the average area covered by spread platelets. Adherent platelet number and area occupied by spread platelets were calculated in 3 random microscopic fields. Data are representative of at least three different experiments and presented as mean ± SEM. Data are analyzed by Student’s paired *t*-test or RM one-way ANOVA with Dunnett’s multiple comparisons test.

Platelets were pre-treated with either longdaysin or D4476, two pharmacologically distinct inhibitors of CK1, or the vehicle, followed by exposure to different physiological agonists. Neither of the inhibitors had any effect on resting (unstimulated) platelets (Supplementary Fig. 1J, K), which was in keeping with lack of adverse effect of longdaysin within 10–50 μM concentration range as reported earlier^11,22,23^. Thrombin (0.25 U/ml) provoked robust platelet aggregation, which was significantly impaired (by 16.76%) in the presence of longdaysin (Fig. 1A, B). CK1 inhibitors, too, restrained platelet aggregation induced by thrombin receptor-activating peptide (TRAP) (Supplementary Fig. 1D, E). Platelet aggregation induced by non-PAR agonists, collagen (2 μg/ml) and ADP (5 μM), was also significantly forestalled in the presence of either longdaysin or D4476 in a concentration-dependent manner (Fig. 1D–M). However, CK1 inhibitors failed to thwart ristocetin (1.25 mg/ml)-induced platelet agglutination (Supplementary Fig. 1F, G), thus ruling out any significant role of casein kinase-1 in driving passive interactions between GP_Ibα_-V-IX and von Willebrand factor.

**Fig. 2:**
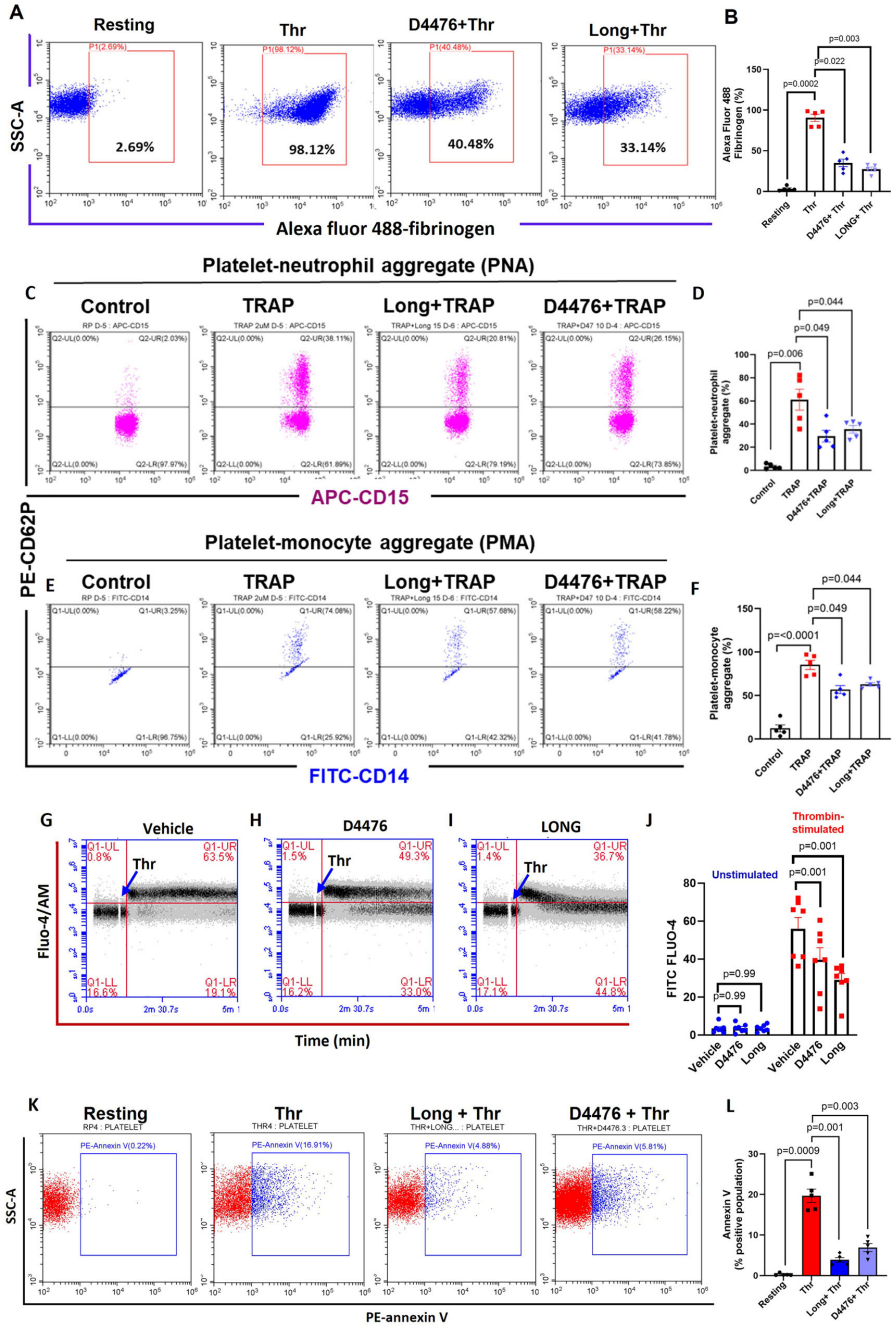
Casein kinase-1 mediates hemostatic responses in agonist-stimulated platelets. **A** Binding of Alexa Fluor 488-conjugated fibrinogen to thrombin-challenged washed human platelets pre-treated with either vehicle or longdaysin or D4476, as indicated. **B** Corresponding bar diagram illustrating the percentage of platelets positive for fluorescently labeled fibrinogen. Flow cytometric analysis of platelet-neutrophil aggregates (**C**, **D**) and monocyte-platelet aggregates (**E**, **F**) in whole blood stained with anti-CD62P-PE (specific for platelets), anti-CD14-FITC (specific for monocytes) and anti-CD15-APC (specific for neutrophil), followed by addition of TRAP (2 μM) in the presence or absence of CK1 inhibitors, as indicated. **G**–**I** Intracellular calcium flux against time in a population of Fluo-4-loaded platelets following exposure to thrombin in the presence or absence of longdaysin and D4476, as indicated. **J** Corresponding bar diagram representing mean of Fluo-4-positive events in platelet populations over a period of 4 min following addition of vehicle or reagents as indicated (*n* = 7). **K**, **L** Phosphatidylserine exteriorization in thrombin (0.25 U/ml)-stimulated platelets. Data are representative of at least three different experiments and presented as mean ± SEM. Results were analyzed by RM one-way ANOVA or two-way ANOVA with Dunnett’s multiple comparisons test or Sidak’s multiple comparisons test.

Aggregation of platelets entails fibrinogen forming bridges between neighboring platelets, as it binds to the ‘active’ conformers of α_IIb_β_3_ integrins on cell surfaces. Thrombin instigated enhanced binding of PAC-1-FITC (antibody that recognizes the open conformation of α_IIb_β_3_) (Fig. 1N, O) and fibrinogen-Alexa Fluor 488 (Fig. 2A, B) to platelet membrane, which were significantly constrained by longdaysin (by 68.54% ± 9.6 and 69.51% ± 4.2, respectively) and D4476 (by 52.40% ± 10.52 and 61.51% ± 6.3, respectively). To further validate these findings, integrin activation was assessed in murine platelets from the extent of fluorescent fibrinogen binding^24,25^. Consistent with the observations in human platelets, thrombin-induced fibrinogen binding to murine cells was markedly diminished in the presence of both inhibitors (Supplementary Fig. 2A, B). These results highlight the conserved nature of regulatory mechanisms controlling integrin activation across species and further underscore the inhibitory potential of longdaysin and D4476 on platelet functions.

Platelets, too, adhere to immobilized fibrinogen through integrins α_IIb_β_3_ that ensues progression to ‘spread’ morphology via sequential formation of filopodia and lamellipodia^26–28^. Keeping with observations above, introduction of longdaysin ensured a drop in the number of platelets adhering to fibrinogen compared to their control counterparts (by 34.88%) (Fig. 1R, S). Extent of platelet spreading (area covered by spread platelets) over immobilized matrix was also found to be significantly impaired (by 50.80% ± 4.68) in the presence of longdaysin (Fig. 1R, T), thus alluding to a vital role of casein kinase-1 in integrin inside-out and outside-in signaling.

Secretion of granule contents is a hallmark of robust platelet activation. Release of adenine nucleotides from dense granules of thrombin-stimulated platelets was significantly abrogated (by 23.35% ± 3.47) in the presence of longdaysin (Fig. 1A, C). Surface externalization of CD62P (P-selectin), a marker for alpha granule release, was also significantly diminished (by 37.56% ± 9.15 and 46.78% ± 5.23, respectively) in platelets pre-treated with either longdaysin or D4476 followed by thrombin exposure (Fig. 1P, Q).

We investigated the impact of CK1 downregulation in thrombin-challenged platelets pre-supplemented with aspirin, an irreversible inhibitor of cyclooxygenase-1. Both PAC-1 binding as well as P-selectin externalization were impaired to a similar extent in aspirin-treated platelets compared to non-aspirinated counterparts (Supplementary Fig. 1A–C), suggestive of minimal or no role of CK1 in thromboxane A2-mediated signaling. ADP released from platelet dense granules, too, contributes to feed-forward activation loops in agonist-activated platelets similar to thromboxane A2. As demonstrated above, CK1 inhibition significantly averted platelet aggregation elicited by ADP (Fig. 1H–K), indicating a key involvement of CK1 in ADP-induced platelet reactivity.

As P-selectin possesses high affinity toward P-selectin glycoprotein ligand-1 (PSGL-1) expressed on leukocyte membranes^29^, we next queried whether inhibition of CK1 activity would compromise platelet-leukocyte interaction. Thrombin receptor-activating peptide (TRAP, 2 µM) prompted a surge in platelet-monocyte as well as platelet-neutrophil aggregate formation (by 6.91 and 18.15 folds, respectively), which were significantly thwarted in the presence of D4476 (by 33.30% ± 7.1 and 51.68% ± 8, respectively) and longdaysin (by 26.35% ± 5.5 and 41.75% ± 6.2, respectively) (Fig. 2C–F). In addition to the p-selectin–PSGL-1 axis, MAC-1 integrin (α_M_β_2_), predominantly expressed on leukocytes, plays a critical role in stabilizing platelet-leukocyte interactions by binding to its ligand GP_Ibα_ on platelets^30–33^. Given the established role of platelet-leukocyte interactions in the pathogenesis of thrombo-inflammatory disease^34–36^, the above observations emphasize a critical role of platelet casein kinase-1 activity in the evolution of ‘thrombogenic’ phenotype.

Reactive oxygen species are critical mediators of platelet activation^37^. To investigate the involvement of casein kinase-1 in redox signaling, we measured cytosolic ROS in thrombin-stimulated platelets following treatment with CK1 inhibitors. Both D4476 and longdaysin significantly attenuated intracellular ROS production by 58.50% ± 4.47 and 76.36% ± 3.83, respectively, thus underlining a seminal role of CK1 in ROS generation driving hemostatic response (Supplementary Fig. 1H, I).

Intracellular Ca^2+^ is a critical player in the regulation of platelet activation and thrombogenicity^38^. Possible role of CK1 in mobilizing intracellular calcium was next analyzed flow cytometrically in Fluo-4-loaded platelets (2 × 10^6^/ml) in the presence of 1 mM calcium within the time lapse 1.5–5.0 min post-thrombin stimulation. Thrombin incited a substantial rise in mean fluorescence of Fluo-4-positive platelets (by 16.09-fold) compared to the pre-stimulation level (Fig. 2G). Percentage of platelets exhibiting fluorescence above the designated threshold (indicated by the horizontal line) surged from 3.48% in unstimulated cells to 56.01% upon agonist challenge (Fig. 2G, upper left and right quadrants, respectively). However, platelets pre-treated with either longdaysin or D4476 exhibited a substantial drop in fluorescence intensity (by 29.01% ± 2.91 and 39.65% ± 2.91, respectively) upon thrombin stimulation (Fig. 2H, I), indicative of slump in cytosolic free calcium, which could contribute to decreased platelet response to agonists.

A sub-population of stimulated platelets externalizes phosphatidylserine (PS) on surface membranes, thus rendering them ‘procoagulant’ at the site of vascular injury^39^. Platelets challenged with thrombin were found to have 69-fold higher PS-expressing procoagulant surface, as assessed from binding of annexin V, which was significantly stymied either by longdaysin (by 80.24% ± 1.545) or by D4476 (by 64.78% ± 1.504) (Fig. 2I, J).

**Fig. 3:**
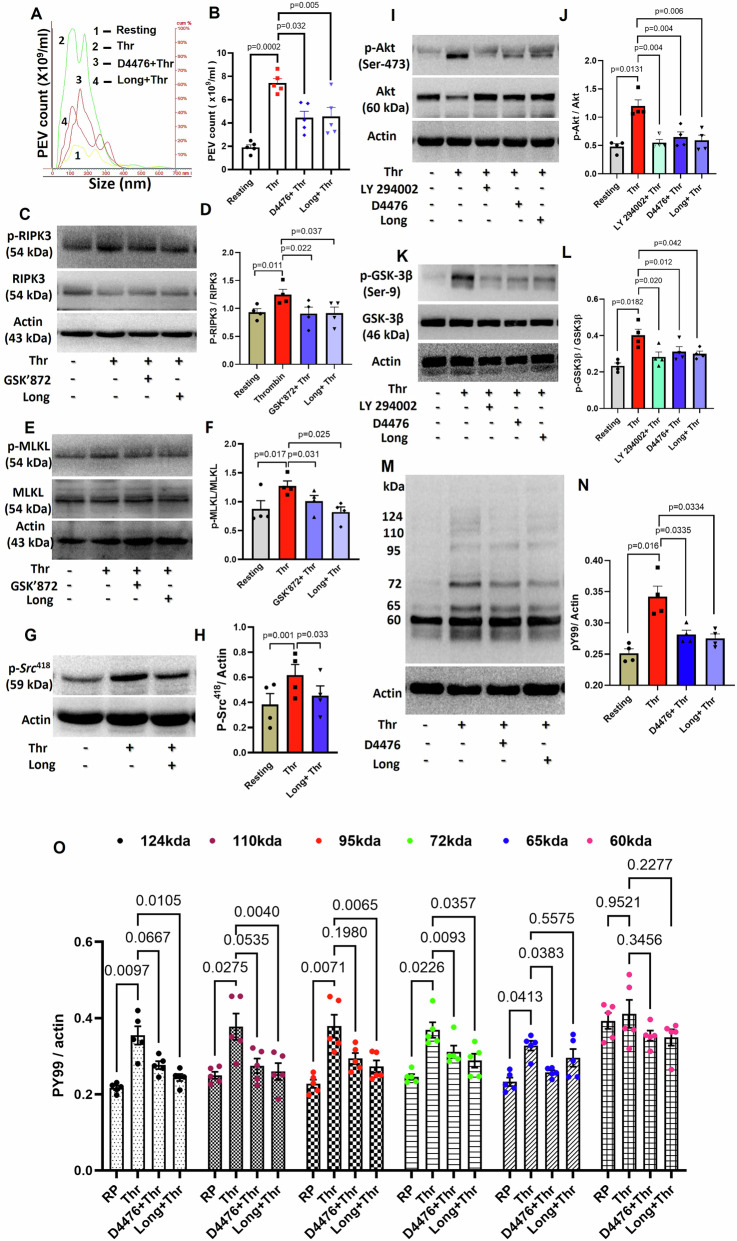
Casein kinase-1 induces PEV release and Tyr/Ser phosphorylation of peptides in agonist-challenged platelets. **A** Nanoparticle tracking analysis (NTA) of count and size distribution of PEVs released from thrombin (0.25 U/ml)-stimulated platelets treated either with vehicle or longdaysin and D4476. **B** Corresponding bar diagram denoting the mean count of released PEVs. **C** Phosphorylation of RIPK3 in platelets pre-treated with either vehicle (DMSO), or GSK’872 (specific inhibitor of RIPK3) (25 μM), or longdaysin (15 μM) as indicated, followed by thrombin (0.25 U/ml) stimulation for 5 min. **D** Corresponding densitometric analysis of p-RIPK3 normalized with RIPK3. **E** phosphorylation of MLKL in platelets pre-treated with either vehicle or GSK’872 or longdaysin, followed by thrombin stimulation as above. **F** corresponding densitometric analysis of p-MLKL normalized with MLKL. **G**, **I**, **K**, **M** Immunoblot analysis demonstrating p-*Src*, p-Akt, p-GSK-3β and global tyrosine-phosphoproteome, respectively, in platelets pre-treated either with vehicle, or longdaysin, or D4476 for 15 min at RT, followed by thrombin (0.25 U/ml) stimulation for 5 min at 37 °C. Molecular weights of peptides with changed states of phosphorylation are indicated on the left. **H**, **J**, **L** Densitometric analysis of p-*Src*, p-Akt, p-GSK-3β, respectively, normalized with their non-phosphorylated peptide counterparts. **N** Densitometric analysis of global tyrosine phosphoproteome normalized with actin. **O** Densitometric analysis of specific tyrosine-phosphorylated peptides, as indicated, normalized with their respective actin bands. Data are presented as mean ± SEM of at least three different experiments. Results were analyzed by RM one-way ANOVA with either Dunnett’s multiple comparisons test or Sidak’s multiple comparisons test.

Stimulated platelets also release extracellular vesicles (PEVs)^40–43^, which are procoagulant in nature, contributing to hemostatic responses^44^. Remarkably, thrombin-induced shedding of PEVs was significantly thwarted in the presence of longdaysin and D4476 (by 38.74% ± 0.42 and 40.1% ± 0.72, respectively) (Fig. 3A, B). In summation, the above observations strongly underline the seminal role played by casein kinase-1 in thrombin-mediated hemostatic responses, which include platelet aggregation, fibrinogen binding to surface integrins, adhesion/spreading, secretion of granule contents, PS exposure, interaction with leukocytes and shedding of extracellular vesicles.

Platelet activation is typified by a significant rise in the pool of tyrosine phosphoproteome attributable to stimulation of *Src* family kinases^45,46^. Thus, exposure to thrombin evoked enhanced phosphorylation of platelet peptides having Mr 60, 65, 72, 95, 110 and 124 kDa (by 4.72%, 40.72%, 50%, 66.5%, 50.98% and 64%, respectively) on tyrosine, which were mitigated in presence of longdaysin (by, 15.13%, 9.8%, 21.87%, 28%, 31.14% and 31%) and D4476 (by, 14%, 21.47%, 15.6%, 22.5%, 27.18%, and 22%, respectively) (Fig. 3M–O: please refer to Supplementary Fig. 4, for corresponding uncropped original blots). Concurrently, thrombin-induced phosphorylation of key signaling molecules, *Src* (Tyr-418), Akt (Ser-473), and GSK-3β (Ser-9), was also significantly abrogated in the presence of the CK1 inhibitors, longdaysin and D4476 (Fig. 3G–L: corresponding uncropped original blots shown as Supplementary Fig. 4), underscoring the crucial role of CK1 in platelet physiology.

In an elegant study, Hanna-Addams and coworkers have demonstrated a fundamental role of CK1 in phosphorylation of RIPK3 and MLKL, the key molecular arbiters governing necroptosis^15^. In another significant report, RIPK3 has been implicated in murine platelet activation and thrombus formation^47^. A more recent study from our laboratory has underlined the critical involvement of MLKL in thrombin (0.5 U/ml) induced platelet reactivity and thrombogenicity^21^. It is, therefore, pertinent here to investigate whether CK1-mediated transition of thrombin-stimulated platelets to functionally active prothrombotic units, as observed in the present study, is orchestrated through the RIPK3-MLKL pathway.

Stimulation of platelets with thrombin (0.25 U/ml) triggered phosphorylation of RIPK3 (at Ser-227) as well as MLKL (at Ser-358), which were significantly abrogated upon pre-treatment with GSK’872, a pharmacological inhibitor of RIPK3, by 27.31% ± 0.05 and 20% ± 0.05, respectively (Fig. 3C–F: The original, uncropped blots corresponding to these results are given in Supplementary Fig. 4). Strikingly, longdaysin, too, brought about significant attenuation in phosphorylation of RIPK3 and MLKL (by 26.73% ± 0.06 and 35% ± 0.08, respectively) in thrombin-challenged platelets (Fig. 3C–F).

Taken together, our findings suggest dual regulatory roles for CK1 in driving platelet activation signaling. CK1 instigates canonical Akt-GSK-3β signaling essential for agonist-induced platelet functional responses. In parallel, it prompts the activation of the RIPK3-MLKL axis, which has been increasingly implicated in the regulation of platelet thrombogenicity.

### Casein kinase-1 governs mitochondrial integrity and platelet bioenergetics

Despite lacking nucleus, platelets typically harbor well-coupled functional mitochondria averaging between 4 and 8 units per cell^48–50^. Studies from our laboratory and others have implicated Warburg’s aerobic glycolysis as well as mitochondrial OXPHOS in sustaining various functional states of platelets, which can be exceedingly energy-intensive^37,48,51–53^. Metabolic plasticity is the key to enabling platelets to meet their extraordinary energy demand with so few mitochondria. As casein kinase-1 emerges to be a critical player in agonist-induced platelet activation, we next queried its contribution to sustaining mitochondrial oxygen consumption rate (OCR) and OXPHOS in resting as well as thrombin-stimulated platelets.

**Fig. 4:**
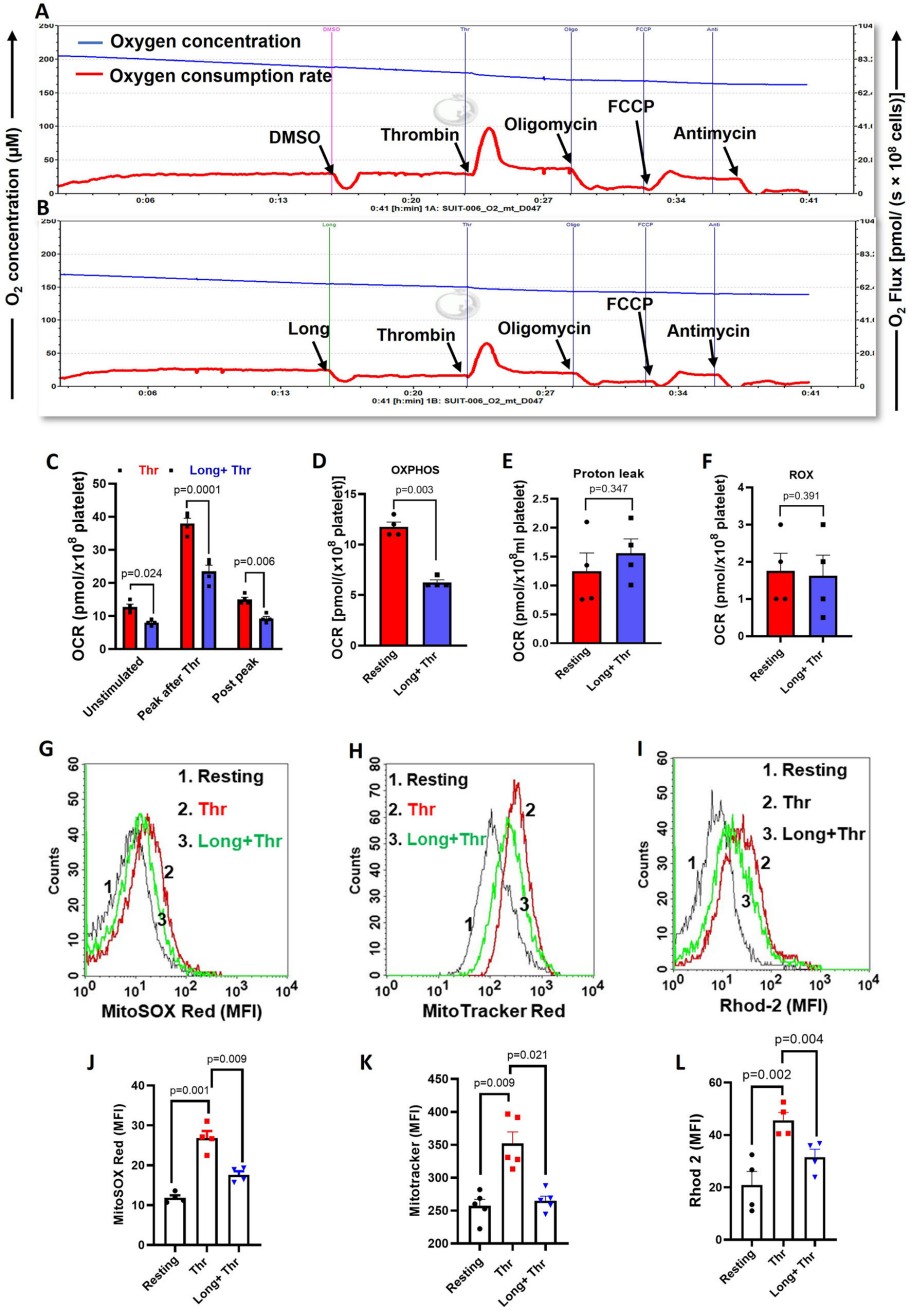
Casein kinase-1 ensures the preservation of functional mitochondria in thrombin-stimulated platelets. **A**, **B** Polarograms representing the rate of oxygen consumption (red trace) and oxygen concentration (blue trace) in thrombin (0.25 U/ml)-stimulated platelets treated either with vehicle (**A**) or longdaysin (15 µM) (**B**). **C**–**F** Corresponding bar diagrams representing routine respiration, ATP-linked respiration (OXPHOS), leak respiration (LEAK) and residual oxygen consumption (ROX) rates in thrombin-stimulated platelets treated with vehicle or longdaysin (15 µM). **G**, **H** Histogram overlays representing fluorescence of MitoSOX Red and MitoTracker Red, respectively, in thrombin (0.25 U/ml)-stimulated platelets pre-incubated with either vehicle or longdaysin (15 µM). **J**, **K** Bar diagrams representing mean fluorescence intensities of MitoSOX Red and MitoTracker Red-positive platelets, respectively. **I** Histogram plots representing fluorescence of Rhod-2-positive cells in platelet papulation pre-incubated with longdaysin followed by the addition of thrombin (0.25 U/ml) in the presence of 1 mM calcium. **L** bar diagram representing the mean fluorescence intensity of Rhod-2-positive cells. Data are representative of at least three different experiments and presented as mean ± SEM. Results were analyzed by Student’s paired *t*-test or RM one-way ANOVA or two-way ANOVA with Dunnett’s multiple comparisons test or Sidak’s multiple comparisons test.

Oxygen flux was monitored in a stirred platelet suspension at high resolution, with samples analyzed at 2 s intervals, employing a Clark amperometric electrode. Prior exposure to longdaysin was observed to minimally affect baseline respiration in resting platelets. However, the surge in oxygen consumption provoked by thrombin (0.25 U/ml) was partially abrogated (by 38% ± 1.6) in platelets treated with longdaysin (Fig. 4A–C), thus underscoring the involvement of CK1 in enabling mitochondrial electron flow. Platelets exposed to oligomycin (1 µg/ml), a specific inhibitor of complex V (ATP synthase) activity, exhibited a significant drop in OCR with negligible leak respiration (proton leak) (Fig. 4A, B, E). OXPHOS embodies coupling between oxygen consumption and ATP synthase activity and is established by eliminating leaks from standard respiration. Significantly, thrombin-triggered oxidative phosphorylation (OXPHOS) displayed a notable decline (by 46.80% ± 0.28) in the presence of longdaysin (Fig. 4A, B, D), accompanied by a concurrent increase in proton leakage. As leak respiration denotes oxygen utilization propelled by proton movement across the inner mitochondrial membrane independent of complex V function, the aforementioned observations overwhelmingly suggest a stabilizing influence of CK1 on the inner mitochondrial membrane that limits proton leakage, thereby maintaining the integrity of well-coupled mitochondria.

The escape of protons from the intermembrane space into the mitochondrial matrix would ensue the collapse of transmembrane potential (ΔΨm), leading to a drop in mitochondrial ROS production^48,54^. Consistent with the above observations on leak respiration, the elevated ΔΨm and mROS in platelet mitochondria evoked by thrombin were significantly blunted upon exposure to longdaysin (by 24% and 34%, respectively) (Fig. 4G–K). Concurrently, ΔΨ_m_-mediated influx of mitochondrial calcium^55^ in thrombin-stimulated platelets was, too, significantly alleviated by longdaysin (by 30.18%) (Fig. 4I, L). Calcium’s crucial role in propelling the tricarboxylic acid (TCA) cycle, accompanied by mitochondrial oxygen consumption^56^, could potentially explain the drop in thrombin-induced spike in OCR in the presence of CK1 inhibitor. Taken together, the above findings suggest that casein kinase-1 reduces proton leak across the inner mitochondrial membrane and thus bolsters mitochondrial respiration through ΔΨ-dependent calcium entry into the matrix.

### Casein kinase-1 governs arterial thrombosis in mice and regulates the generation of platelet thrombi ex vivo

**Fig. 5:**
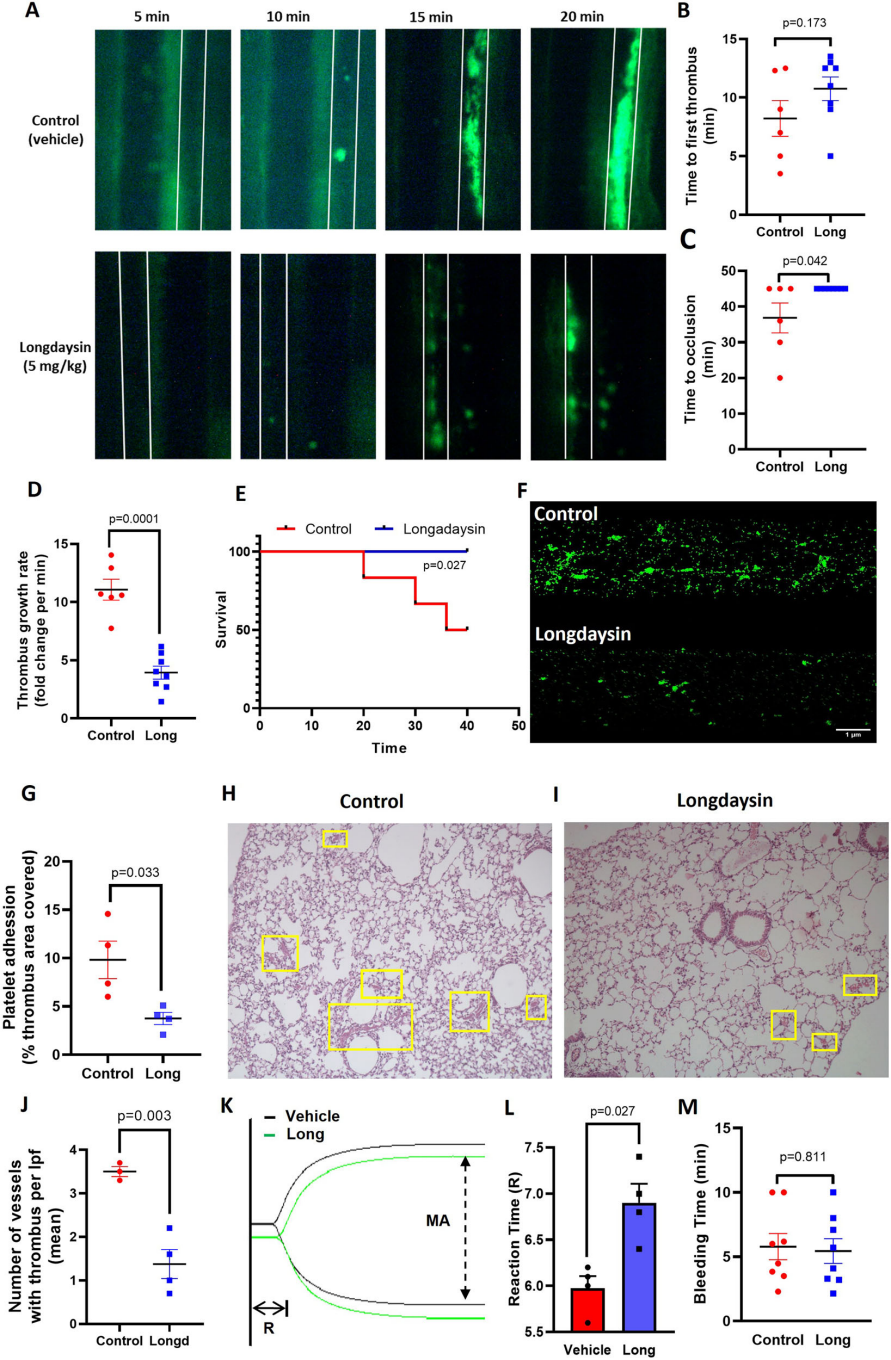
Inhibition of casein kinase-1 activity impedes arterial thrombosis and pulmonary thromboembolism in mice, and restricts formation of platelet thrombi upon immobilized collagen. **A** Time-lapse images illustrating mesenteric arteriolar thrombosis in mice, pre-administered with either vehicle (control) or longdaysin (5 mg/kg), captured 5, 10, 15 or 20 min following ferric chloride-induced injury of the mesenteric arterioles. **B**–**D** Scatter dot plots representing time to first thrombus formation (**B**), time to occlusion (**C**) and thrombus growth rate (**D**). **E** Kaplan–Meier curve exhibiting percent survival at various time points of observation in mice pre-administered with either vehicle (*n* = 6) or longdaysin (*n* = 8). **F** Confocal images exhibiting growth of platelet thrombi over collagen at 8 min following start of perfusion (at shear rate 1500 s^−^^1^) in control (DMSO) as well as longdaysin (15 μM)-pre-treated cells. **G** Corresponding bar diagram representing total surface area covered by platelet thrombi after 8 min perfusion over collagen matrix, studied in 4 representative fields (*n* = 4). **H**, **I** Light microscopy images (×10 magnification) of hematoxylin and eosin-stained lung sections from mice administered with either DMSO (control) or longdaysin (5 mg/kg), followed by collagen (1 mg/kg) and epinephrine (100 μg/kg). White arrows indicate thrombi within the lumen of pulmonary vessels. **J** Scatter dot plots illustrate the number of thrombosed pulmonary vessels per low-power field in mice treated with either vehicle (control) (*n* = 3) or longdaysin (*n* = 5). **K** Thromboelastogram of kaolin-stimulated citrated whole blood pre-incubated with either vehicle (black tracing) or with longdaysin (25 µM) (green tracing). **L** Corresponding bar diagram representing reaction time (R) reflective of initiation of fibrin formation (*n* = 4). **M** Scatter dot plots showing tail-bleeding times in mice pre-treated with either vehicle (control) or longdaysin (*n* = 8). Data are representative of at least three individual experiments (mean ± SEM). Data are analyzed by either unpaired (**B**–**D**, **J**, **M**) or paired (**G**, **L**) Student’s *t*-test (unpaired for in vivo and paired for in vitro and ex vivo).

Platelets play a critical role in the development of arterial thrombosis, which is central to emergencies such as acute myocardial infarction and ischemic stroke. To investigate the involvement of CK1 in the generation of occlusive intramural thrombi in vivo, we tested the effect of longdaysin in a murine model of mesenteric arteriolar thrombosis. Platelets were fluorescently labeled, and mice were intraperitoneally administered with either longdaysin (5 mg/kg) or vehicle (DMSO). Intramural thrombus was induced by topical application of 20% ferric chloride for 3 min on exteriorized mesenteric arterioles. Thrombus formation was visualized in real time by intravital imaging employing an epifluorescence video microscope equipped with a high-speed camera (Fig. 5A).

We measured the time to first thrombus formation, the rate of thrombus growth and the time till complete vessel occlusion as indicators of the initiation, propagation and stabilization of thrombus, respectively. Remarkably, mice administered with longdaysin exhibited significantly delayed thrombus formation compared to vehicle-treated animals (average times to form first thrombus: control, 8.21 min; longdaysin, 10.75 min) (Fig. 5B). While the mean occlusion time for control mice was 36.83 min, stable occlusion failed to occur even after 40 min from the time of injury in all longdaysin-pre-treated mice (Fig. 5C). Longdaysin also significantly impeded the growth rate of the thrombus (by 64.41% ± 1.006) (Fig. 5D) and probability of survival (Fig. 5E). The Kaplan–Meier analysis and the log-rank test determined the time to arterial occlusion (Fig. 5E; Supplementary Video 1, 2). They provided robust evidence in support of a greater percent of survival observed over time in longdaysin-administered mice that contributed to the prolonged survival of this group of animals compared to control counterparts. These findings suggest that CK1 in platelets plays a crucial role in the initiation and propagation of arterial thrombosis in vivo.

In order to explore the role of casein kinase-1 in the generation of thrombus ex vivo, we next studied dynamic adhesion of human platelets and thrombus formation on immobilized collagen under arterial shear (1500 s^−^^1^) employing the BioFlux microfluidics platform. Washed human platelets as well as PRP were individually pre-incubated with either of the inhibitors, longdaysin D4476, or the vehicle, for 10 min, and allowed to superfuse over a collagen-coated surface at the specified shear for 10 min to enable thrombus generation. Strikingly, prior exposure to longdaysin significantly constrained total surface area covered by platelet thrombi (by 61.77%) in washed platelets (Fig. 5F, G). Similarly, PRP dually stained with Calcein green and PE-CD62P also exhibited a significant drop in thrombus formation upon prior exposure to longdaysin (by 49.37%, ±4.5). However, a nonsignificant difference was observed in the presence of D4476 (by 33.54%, ±5.5) (Supplementary Fig. 2I, J), which underscores a fundamental role of casein kinsase-1 in the regulation of platelet-mediated thrombogenicity.

To further corroborate these findings, we employed a mouse model of collagen-epinephrine-induced pulmonary thromboembolism. Hematoxylin and eosin-stained lung sections from longdaysin-treated mice revealed significantly fewer thrombosed pulmonary vessels than their control counterparts (longdaysin, 1.37 per low-power field or lpf; control, 3.5/lpf) (Fig. 5H–J), which further validated the above observations on the role of CK1. In parallel, platelet function assays were performed to investigate the underlying mechanisms, focusing on integrin activation and intracellular calcium flux in platelets isolated from mice intravenously pre-administered with vehicle or longdaysin (5 mg/kg). Consistent with in vivo findings, thrombin exposure ex vivo markedly enhanced fibrinogen binding and intracellular free calcium in platelets from vehicle-treated mice (by 44.53- and 11-fold, respectively) (Supplementary Fig. 2C, E, F, H, respectively), which were significantly attenuated following longdaysin administration (Supplementary Fig. 2D, E, G, H, respectively).

In keeping with the above, we next analyzed the contribution of CK1 on intrinsic pathway of blood coagulation by employing kaolin-activated thromboelastography. Reaction time (R) denotes the time of latency from the start of the test to initial fibrin formation. Pre-treatment of platelets with longdaysin significantly prolonged the reaction time from 5.9 to 6.9 min (Fig. 5K, L), which reflected delayed kinetics of thrombus formation through CK1 inhibition. We also evaluated the effect of longdaysin on the extrinsic pathway of coagulation employing the Prothrombin Time (PT) assay, as well as thromboelastography of re-calcified blood; however, no significant change either in PT or R value was detected (Supplementary Fig. 2K–M). Thus, observations from the in vivo murine model of thrombosis, as well as thromboelastography, underscored an indispensable role of CK1 in determining thrombus stability.

Subsequently, we investigated the impact of longdaysin on primary hemostasis in mice employing tail-bleeding assay. Administration of longdaysin (5 mg/kg) did not affect the bleeding time (time required until the cessation of bleeding for ≥10 s) when compared to vehicle-treated control mice (Fig. 5M), thus emphasizing the lack of potential bleeding complications associated with the drug.

To evaluate the impact of CK1 inhibition on platelet viability, platelets were incubated with varying concentrations of the longdaysin (5, 10, 20, and 30 µM) or longdaysin with thrombin (0.25 U/ml) and subjected to MTT assay. We observed no significant alteration in the number of viable cells under above experimental conditions (Supplementary Fig. 1L). Thus, longdaysin effectively prohibits agonist-induced platelet activation and reduces thrombogenic potential without compromising platelet viability or inducing cytotoxic effects.

## Discussion

Employing longdaysin and D4476, two pharmacologically independent cell-permeable inhibitors of α and δ isoforms of CK1^11,22,57^, this study reveals a yet unrecognized fundamental role of CK1 in fueling agonist-induced platelet activation that include cell-cell aggregate formation, integrin activation, secretion of granule contents, shedding of extracellular vesicles, spreading on immobilized matrix, leukocyte-platelet interactions, raised intracellular calcium as well as tyrosine phosphoproteome, generation of procoagulant surface, and thrombus formation under arterial shear. These findings align with the broader role of casein kinases in cellular dynamics, as CK2 is known to regulate focal adhesion restructuring through phosphorylation of focal adhesion kinase (FAK)^58,59^. Notably, platelet adhesion and spreading involve signaling cascades initiated by PARs, which couple to G_12/13_- and G_q_-linked pathways. The G_12/13_ drives RhoA-mediated actomyosin contractility, enabling cytoskeletal remodeling essential for shape change and spreading^60–62^.

Further insights into CK1’s involvement in the generation of procoagulant platelet sub-population can be drawn from elegant studies by Agbani et al.^63,64^ that demonstrate morphological transformation of platelets characterized by membrane ballooning, a process mediated by aquaporins. Future studies should explore the interplay between CK1 signaling and aquaporin-mediated membrane dynamics to attribute their roles in thrombogenicity. Platelet procoagulant membrane is essential for the assembly of clotting factors sourced from both intrinsic as well as extrinsic pathways^65,66^. Longdaysin restrains platelets from adopting a procoagulant surface upon stimulation with thrombin (Figs. 2K, 3A). This would lead to impaired thrombin generation and delayed formation of fibrin-rich clot, as reflected from significantly prolonged Reaction Time (R) in kaolin-induced thromboelastogram representing the intrinsic pathway of coagulation^67^. However, there was no significant change in Prothrombin Time in longdaysin-treated samples that could be attributable to differential kinetics of assembly of extrinsic tenase (Xase) complex on platelet surface compared to its intrinsic counterpart.

Overall, the agonist-induced platelet activation and aggregation processes rely heavily on ATP supplied by mitochondrial oxidative phosphorylation (OXPHOS)^68,69^. Keeping with this, we observed that CK1 inhibition, too, prompted impairment in mitochondrial OXPHOS, accompanied by dissipation of transmembrane potential (ΔΨ_m_), augmented proton leak and drop in both mitochondrial calcium as well as mROS, thereby underscoring a key role of CK1 in preservation of inner mitochondrial membrane integrity. By maintaining the well-coupled state of mitochondria, CK1 ensures mitochondrial ATP production in stimulated platelets, which are essential for platelet activation responses to agonist challenge^48^. Hence, CK1 plays a critical role in stabilizing mitochondrial function, ensuring a consistent energy supply necessary for platelet responses to agonists.

The role of CK1 in the phosphorylation of RIPK3 and MLKL, molecules that govern necroptosis, has already been acknowledged in HeLa cells^15^. In a more recent report, we have established a causal link between MLKL phosphorylation and induction of platelet activation and thrombogenesis^21^. Consistent with the above observations, the present study demonstrates that inhibition of CK1 activity in thrombin-treated platelets blunted phosphorylation of RIPK3 and MLKL, and restrained platelet activation, thus offering a mechanistic basis linking the CK1-RIPK3-MLKL axis to agonist-induced thrombogenic responses. Furthermore, by maintaining the well-coupled state of mitochondria, CK1 sustains platelet energy metabolism and ATP production essential for platelet activation responses to agonist challenge^48^.

CK2β has been shown to participate in thrombopoiesis, platelet activation, and arterial thrombosis in vivo^70,71^. Glycogen synthase kinase-3β (GSK-3β) is another serine/threonine kinase that plays a central role in Wnt/β-catenin developmental signaling^72^. It cooperates with CK1 as a member of the multiprotein ‘degradation complex’ leading to phosphorylation and proteasomal cleavage of β-catenin^73^. GSK-3β remains active in unstimulated platelets, while its activity is attenuated upon phosphorylation by Akt kinase following agonist exposure^74^. Thus, GSK-3β, unlike CK1, acts as a negative regulator of platelet activity and thrombosis^3,75–77^. In line with the above observations, both longdaysin as well as D4476 significantly constrained phosphorylation of GSK-3β and Akt in thrombin-stimulated platelets. Thus, inhibiting CK1 could serve as a potential therapeutic strategy to thwart platelet activation and thrombotic responses by targeting the Akt-GSK-3β signaling axis.

In summary, this study presents compelling evidence in support of a central role of CK1 in orchestrating agonist-induced platelet activation progressing to prothrombotic phenotype. The existing antiplatelet regimens are commonly plagued by bleeding complications. Remarkably, inhibition of CK1 did neither impair the viability of platelets nor impact primary hemostasis, while it significantly retarded thrombosis as evident from the in vivo and ex vivo studies. Thus, CK1 may potentially serve as a promising target for therapeutic intervention against thrombotic disorders.

## Methods

### Materials

Anti-MLKL polyclonal antibody and anti-phospho (Ser-358)-MLKL (p-MLKL) monoclonal antibody were the products of GeneTex (#GTX107538) and Abcam (#ab187091), respectively. Monoclonal antibodies against phospho-RIPK3 (Ser-227) (#93654), RIPK3 (# 13526), phospho-AKT (Ser-473) (#4051), AKT (#9272), phospho-GSK-3β (Ser-9) (#9336), and GSK-3β (#12456) were from Cell Signaling Technology. Antibody against phosphotyrosine (pY99, #sc-7020) was procured from Santa Cruz Biotechnology. PE-mouse anti-human CD62P (#550561), FITC-mouse anti-human PAC-1 (#340507), FITC-mouse anti-human CD14 (#555397), APC-mouse anti-human CD15 (#551376) were from BD Biosciences. Anti-phospho-*Src* (Tyr-418) monoclonal antibody (SC1T2M3) was the product of Invitrogen. Anti-actin antibody (#A2066), longdaysin (#SML0127), thrombin (#T6884), RIPK3 Inhibitor (GSK’872) (#530389), ethylene glycol tetraacetic acid (EGTA), ethylenediaminetetraacetic acid (EDTA), Acetylsalicylic acid (A5376), H2DCFDA (#D6883), thrombin receptor-activating peptide (TRAP) (#S1820) and DMSO were from Sigma. D4476 (HY-10324) was from MedChemExpress. PE-labeled annexin V (#640908) was procured from BioLegend. Chrono-lume luciferin luciferase reagent (#395), ADP (#384), ristocetin (#396) and collagen (#385) were products of Chrono-log. HEPES (#391340), polyvinylidene fluoride (PVDF) membrane (#IPVH00010) and Immobilon Western chemiluminescent HRP substrate (#WBKLS0100) were from Millipore. Bovine serum albumin (#MB083) and MTT (3-(4,5-dimethylthiazol-2-yl)-2,5-diphenyltetrazolium bromide) (# RM1131) was from HiMedia. MitoTracker Red (#M7512), MitoSOX Red (#36008), Alexa fluor 488-conjugated phalloidin (#A12379), Rhod-2/AM (#R1244), fibrinogen (Alexa fluor 488-conjugated) (#F13191), goat-anti-rabbit IgG (Alexa fluor 488-conjugated) (#A11008), Fluo-4/AM (#F14201) and Restore Western Blot Stripping Buffer (#21059) were from Invitrogen. HRP-conjugated goat anti-rabbit and anti-mouse IgG were from Bangalore Genei. Triton X-100, Tween-20, CaCl_2_ and reagents for electrophoresis were purchased from Merck. Cell Viability Assay Kit (CellTiter-Glo) (#G7570) was the product of Promega. Prothrombin Time reagent (STA-NeoPTimal-5, #01163) was from Stago. The remaining chemicals were of analytical grade. Type I deionized water (18.2 MΩ.cm, Millipore) was used for the preparation of solutions.

### Platelet isolation

Platelets were isolated from fresh human blood by differential centrifugation^21,78,79^. In brief, peripheral venous blood was collected in acid citrate dextrose (ACD) tubes and centrifuged at 100×*g* for 20 min to isolate platelet-rich plasma (PRP). The PRP was then centrifuged at 800×*g* for 7 min to pellet the platelets, supplemented with 1 µM PGE1 and 2 mM EDTA. The platelet pellet was washed with buffer A (20 mM HEPES, 134 mM NaCl, 2.9 mM KCl, 1 mM MgCl_2_, 0.34 mM NaH_2_PO_4_, 12 mM NaHCO_3_; pH 6.2) containing 5 mM glucose, 0.35% BSA, and 1 µM PGE1. Finally, platelets were then resuspended in buffer B (same composition as buffer A, at pH 7.4) with 5 mM glucose, and the count was adjusted to 2–4 × 10^8^ cells/ml using an automated cell counter (Multisizer 4, Beckman Coulter). Whenever required, aspirinated-washed platelets were isolated as described previously^20,37^. Briefly, PRP was collected and incubated with 1 mM acetylsalicylic acid for 15 min at 37 °C. Subsequently, EDTA (5 mM) was added to the PRP, which was then centrifuged at 800×*g* for 10 min. The resulting pellet was washed with Tyrode buffer containing EGTA and finally resuspended in physiological buffer B. Leukocyte contamination was less than 0.015%. All steps were executed under sterile conditions, and precautions were taken to maintain the cells in a resting state.

### Platelet aggregation

Washed human platelets or platelets suspended in plasma (PRP), pre-treated either with longdaysin or D4476 or vehicle (DMSO), were stirred at 1200 rpm in a Chrono-log optical lumi-aggregometer (Model 700-2) at 37 °C for 5 min following the addition of the agonist. Platelet aggregation was recorded as the percentage change in light transmission over time, whereas 100% represents maximum light transmission through a platelet-free buffer B.

### Secretion of platelet granule contents

Surface expression of P-selectin (CD62P) was assessed as a marker of α-granule secretion in platelets^68^. Briefly, resting or thrombin-stimulated (0.25 U/ml) platelets were incubated with FITC-labeled anti-CD62P antibody (5% v/v) for 30 min at room temperature in dark, without agitation. Following staining, the samples were resuspended in sheath fluid and analyzed using a FACSCalibur flow cytometer (BD Biosciences). Forward scatter and side scatter voltages were set to E00 and 350, respectively, with a threshold of 52 V. A gated region was defined to encompass individual platelets, effectively excluding noise and other nonspecific events. Fluorescence data were acquired with logarithmic amplification in four quadrants, collecting 10,000 events within the platelet gate for each sample, and analyzed using CellQuest Pro software.

Secretion of adenine nucleotides from platelet dense granules was measured by adding 15 μl of Chrono-lume reagent (2 μM luciferase/luciferin stock concentration) before agonist stimulation. The luminescence generated was monitored in a lumi-aggregometer simultaneously with platelet aggregation.

### Study of platelet integrin α_IIb_β_3_ activation and fibrinogen binding

Platelet activation induces a conformational change in surface α_IIb_β_3_ integrins, converting them into an open state that facilitates high-affinity binding to fibrinogen^80^. To assess integrin activation, washed human platelets (2.5 × 10⁸/ml) were pre-treated with either Longdaysin, or D4476, or vehicle, followed by stimulation with thrombin (0.25 U/ml) for 5 min at 37 °C. Platelets were then stained with FITC-labeled PAC-1 antibody (5% v/v), which specifically recognizes the active form of αIIbβ3, or incubated with Alexa Fluor 488-conjugated fibrinogen (10 μg/ml) to detect fibrinogen binding. After 30 min of staining, the samples were diluted with sheath fluid and analyzed by flow cytometry.

### Measurement of annexin V binding

Phosphatidylserine (PS) externalization, a key marker of procoagulant platelets, was assessed via annexin V binding, as previously^81^. Washed human platelets were pre-incubated with CK1 inhibitors, or vehicle for 15 min, followed by stimulation with thrombin (0.25 U/ml) for 5 min at 37 °C under static conditions. PE-conjugated annexin V (0.2% v/v) and 2 mM CaCl₂ were subsequently added to the samples, which were incubated for 30 min in the dark at room temperature. After incubation, the degree of annexin V binding, indicative of phosphatidylserine exposure, was quantified using flow cytometry.

### Immunoblotting

Platelet proteins were separated on 10% SDS-PAGE (sodium dodecyl sulfate polyacrylamide gel electrophoresis) gels and electrophoretically transferred onto polyvinylidene difluoride (PVDF) membrane by employing Trans-Blot Turbo Transfer System (Bio-Rad) at 20 V/1.3 A for 20 to 25 min. Nonspecific protein binding sites were blocked with 5% non-fat dry milk in Tris-buffered saline (10 mM Tris-HCl and 150 mM NaCl, pH 8.0) with 0.05% Tween-20 (TBST) for 1 h at RT. Following 3 washing with TBST for 5 min each, membranes were incubated overnight at 4 °C with following primary antibodies at specified dilutions under gentle agitation: p-MLKL (1:1000), MLKL (1:1000), p-RIPK3 (1:800), RIPK3 (1:800), p-Y^99^ (1:1200), p-Src^418^ (1:1000), p-Akt (1:1250), Akt (1:1250), p-GSK-3β (1:1250) and GSK-3β (1:1250), and β-actin (1:5000). After similar washing steps, blots were incubated with either HRP-conjugated anti-rabbit or anti-mouse IgG secondary antibodies (1:5000) for 1 h at RT. Antibody binding was detected using an enhanced chemiluminescence detection kit (Millipore). Images were acquired on a multispectral Bio Spectrum 800 imaging system (UVP) and quantified using VisionWorks LS software (UVP).

### Analysis of leukocyte-platelet interaction in whole blood

Leukocyte-platelet interaction was measured as described earlier^21,29,82^. A cocktail containing 2 µl each from PE-anti-CD62P (platelet-specific), APC-anti-CD15 (neutrophil-specific) and FITC-anti-CD14 (monocyte-specific) antibodies was added to fresh human blood (25 µl) and mixed gently. Sample was incubated either with vehicle (DMSO, 0.25%) or longdaysin (25 µM) or D4476 (20 µM) for 10 min. Platelet-leukocyte interaction was induced upon addition of TRAP (2 μM) for 10 min at RT. Red blood cells were lysed with 800 μl FACS lysis solution (1X, BD Biosciences) for 10 min at RT. The neutrophil and monocyte populations were gated employing specific antibodies. A quadrant dot plot of FITC-CD14 versus APC-CD15 fluorescence was generated using the CytExpert Software (2.4.0.28). Neutrophil and monocyte populations can also be identified employing amorphous gates for monocyte (high fluorescence and low SSC) and for neutrophil (low fluorescence and high SSC) as described in previous reports^21,29,48,82^. Platelet population within the gating region was identified from PE-CD62P fluorescence, as indicated (Supplementary Fig. 3). All fluorescence data were acquired employing 4-quadrant logarithmic amplification for 10000 events in either neutrophil or monocyte gate from each sample and analyzed using CytExpert Software.

### Isolation and analysis of platelet-derived extracellular vesicles (PEVs)

PEVs were isolated and characterized as described previously^21,42^. Platelets were pre-incubated either with Longdaysin or D4476, followed by stimulation with thrombin (0.25 U/ml). The cells were centrifuged at 800×*g* for 10 min, and subsequently at 1200×*g* for 2 min at 22 °C to obtain platelet-free supernatants containing platelet extracellular vesicles (PEVs). The PEV-containing supernatants were diluted 10-fold in Buffer B prior to analysis. Nanoparticle Tracking Analysis (NTA) was performed, wherein a beam from a solid-state laser source (635 nm) was directed through the sample to quantify and characterize the PEVs. Light scattered by rapidly moving particles in suspension in Brownian motion was observed under a 20X microscope. This analysis revealed hydrodynamic diameters of the particles, calculated using the Stokes-Einstein equation, ranging from 10 nm to 1000 nm, with concentrations between 10⁷ and 10⁹ particles/ml. The movement of each extracellular vesicle (EV) in the x and y directions was tracked, with the average displacement captured by a CCD camera at a frame rate of 30 frames per second, attached to the microscope. Both capture and analysis were performed using NanoSight LM10 (Malvern) and NTA 2.3 analytical software, which provide an estimate of particle size and counts in the sample.

### Evaluation of platelet intracellular free calcium [Ca^2+^]_i_ using Fluo-4/AM

Washed platelets (2 × 10^6^/ml) were incubated with Fluo-4/AM for 30 min at RT in dark. Following appropriate gating of platelets, events were analyzed in the FL1 channel of flow cytometer (BD Biosciences, model Accuri C6+) within the time lapse of 1.5 to 5.0 min (located in the upper right quadrant). Baseline calcium levels were monitored for 60 s, followed by the addition of thrombin (0.2 U/ml)^21,38,83^.

### Prothrombin time assay

Prothrombin time (PT) assay for evaluating the extrinsic coagulation pathway is based on the principle that tissue factor, in the presence of calcium ions, activates factor VII, which subsequently activates factor X, leading to the conversion of prothrombin (factor II) to thrombin, and ultimately resulting in fibrin clot formation^84^. In this assay, citrated whole blood was centrifuged at 2000×*g* for 10 min to obtain platelet-poor plasma. Typically, 100 µl plasma was mixed with 200 µl pre-warmed thromboplastin reagent (containing tissue factor and calcium chloride to initiate clotting), and the time (s) taken for clot formation was measured with a semi-automated viscosity-based coagulation analyzer (Haemostasis Analyzer, Stago *STart*)^85^.

### High-resolution mitochondrial respirometry

Mitochondrial respiration was assessed utilizing a high-resolution respirometer (Oxygraph-2k, Oroboros Instruments) at 37 °C under stirring conditions (750 rpm). Washed human platelets (1.5 × 10^8^/ml, in buffer B supplemented with 5 mM glucose) were introduced into the oxygraph chamber. Initially, respiration was allowed to stabilize at the routine state, representing the physiological coupling state regulated by cellular energy demands for oxidative phosphorylation. Subsequently, platelets were exposed to either the CK1 inhibitor (longdaysin) or DMSO (vehicle) for 5 min, followed by the addition of thrombin (0.25 U/ml). Changes in oxygen flux were continuously monitored in real-time at high resolution (sampling at 2 s intervals). Oxidative phosphorylation (OXPHOS) and leak respiration were quantified subsequent to the introduction of oligomycin (1 μg/ml), while residual oxygen consumption (ROX) was calculated after the addition of antimycin (1 μg/ml) to the cell suspension. Air saturation calibration was conducted daily before commencing experiments by allowing Millipore water or buffer B to stir with air in the oxygraph chamber until equilibrium was achieved with the generation of stable signal. All experiments were carried out at an oxygen concentration in the range of 100–205 μM O_2_. Data acquisition and analysis were performed utilizing DatLab 7.4 software provided by Oroboros Instruments^37,86^.

### Quantification of mitochondrial transmembrane potential (Δψ_m_)

Washed human platelets (2.5 × 10⁸/ml) were pre-treated with Longdaysin, or vehicle, followed by stimulation with thrombin (0.25 U/ml) for 5 min at RT. Platelets were then stained with MitoTracker Red (500 nM) for 20 min at room temperature. Fluorescence from the labeled platelets was analyzed by flow cytometry (BD Biosciences, FACSCalibur).

### Quantification of mitochondrial reactive oxygen species (mROS)

Mitochondrial superoxide generation in platelets was evaluated as described previously^21^. In brief, platelets pre-treated with the inhibitor or vehicle stimulated with thrombin (0.25 U/ml) for 5 min at RT. The platelets were then stained with MitoSOX Red (5 μM) for 20 min at RT in the dark. Mitochondrial ROS levels were analyzed in the labeled platelets employing flow cytometry (BD Biosciences, FACSCalibur).

### Flow cytometric analysis of mitochondrial calcium flux

Platelets (2.5 × 10^8^/ml), pre-treated with Longdaysin or vehicle, were stimulated with thrombin (0.25 U/ml) for 5 min, followed by incubation with the mitochondrial calcium indicator Rhod-2/AM (5 μM) for 30 min in the dark at RT. Mitochondrial calcium flux, as indicated by changes in Rhod-2 fluorescence, was measured using flow cytometry (BD Biosciences, FACSCalibur).

### Platelet adhesion and spreading

Glass slides were first cleaned and coated with fibrinogen (100 µg/ml) for 1 h, followed by blocking with 100 µl bovine serum albumin (10 mg/ml) for 1 h. The slides were then washed three times with 1X PBS. Washed platelets (2 × 10⁷ cells/ml), pre-treated with either longdaysin or vehicle for 20 min, were overlaid onto the fibrinogen-coated slides for 30 min at room temperature. The cells were fixed with 2% paraformaldehyde (PFA) for 20 min, followed by three washes with 1X PBS. Platelets were then permeabilized with 0.02% Triton X-100 for 1 min, washed, and stained with Alexa Fluor 488-conjugated phalloidin (1 µM) for 15 min. Adherent cells were examined using a Zeiss LSM 700 laser scanning confocal microscope with a ×63 objective and a pinhole size of 1 AU. Images were acquired using ZEN imaging software and further analyzed using ImageJ (National Institutes of Health). Four to five distinct fields were captured for each slide^20^.

### Platelet thrombus formation on immobilized collagen matrix under arterial shear

Platelet adhesion and thrombus growth on immobilized collagen matrix were quantified employing BioFlux (Fluxion Biosciences) microfluidics system as described previously^79^. Wells of high-shear plates were coated with 50 µl of collagen (100 µg/ml) at 10 dynes/cm² for 30 s and incubated for 1 h at RT. Wells were blocked with 1% bovine serum albumin at 10 dynes/cm² for 15 min at room temperature. Calcein green-stained platelets (2 µg/ml) were allowed to perfuse over collagen at a physiological arterial shear rate of 1500 s⁻¹ for 5 min. In different experiments, platelets suspended in plasma (PRP), dually stained with Calcein green and PE-CD62P, were also similarly superfused over collagen-coated matrices. Platelet adhesion and thrombus formation were recorded in a fixed field over time, with representative images captured from 5–10 different fields. The total thrombus area at 5 min was analyzed in 4 representative fields using ImageJ software (NIH).

### Study of intravital imaging of thrombus formation in murine mesenteric arterioles

Thrombus formation in murine mesenteric arterioles induced by ferric chloride was imaged using intravital microscopy as already described^37,79^. Briefly, male and female Swiss albino mice, aged 4–5 weeks and weighing 8–10 g each, were intraperitoneally administered with either longdaysin (5 mg/kg) or vehicle (DMSO). After an interval of 30 min, mice were anesthetized with a ketamine/xylazine cocktail (100 mg/kg ketamine and 10 mg/kg xylazine) administered intraperitoneally. Circulating platelets were fluorescently labeled by injecting DyLight 488-labeled anti-GPIbβ antibody (0.1 µg/g body weight) diluted in 50 μl sterile PBS into the retro-orbital plexus of the mice. Mesenteric arterioles of diameter 100–150 μm were isolated and focused under epifluorescence inverted video microscope (Nikon model Eclipse Ti-E) equipped with a monochrome CCD cooled camera. Thrombosis was induced in the exteriorized arteriole by topically applying a Whatman filter paper saturated with 20% ferric chloride solution for 3 min, and real-time thrombus formation was monitored. Thrombus formation was recorded by using a high-speed camera for 40 min or until occlusion. Subsequent analysis of the recorded movies using Nikon image analysis software (NIS Elements) allowed for the determination of key parameters: (a) time to formation of the first thrombus (>20 µm in diameter), (b) time to vessel occlusion (defined as the duration from injury until blood flow cessation for 30 s), and (c) thrombus growth rate (growth of thrombi >30 µm in diameter over period of 3 min). The fold increase in thrombus diameter was calculated by dividing the diameter of the thrombus at a given time (n) by the diameter of the same thrombus at time (0) (defined as the moment when the thrombus reached approximately 30 µm in diameter).

### Pulmonary thromboembolism

Pulmonary thromboembolism (PTE) was induced in 18 to 20 g Swiss albino mice of either sex as already described^37,87^. Mice were administered vehicle (DMSO) or longdaysin (5 mg/kg) via intraperitoneal injection for 30 min, followed by anesthesia with a ketamine/xylazine cocktail (100 mg/kg ketamine and 10 mg/kg xylazine). Pulmonary embolism was induced by intravenous administration of a collagen (1 mg/kg) and epinephrine (100 μg/kg) cocktail via the retro-orbital plexus. The mice that survived were euthanized after observation for a maximum of 20 min by cervical dislocation. Lungs were perfused with 1× PBS, dissected out, and immediately fixed in 10% neutral buffered formalin for 24 h. Histological sections were prepared from paraffin-embedded lung tissues and stained with hematoxylin and eosin (H&E). Thrombus presence in the pulmonary vessels was examined using a Nikon Eclipse Ti-E light microscope. For each sample, at least 5 low-power fields (lpf) (×10 magnification) were observed to assess the number of vessels occluded by thrombi. The count of thrombosed pulmonary vessels per lpf was determined in pulmonary sections from each mouse.

### Thromboelastography (TEG)

Hemostatic parameters in human blood were investigated with the help of Thromboelastograph 5000 Hemostasis Analyzer System (Haemonetics) and TEG analytical software. Citrated whole human blood (1 ml) was incubated with CK1 inhibitor or vehicle for 15 min at RT, followed by transfer to kaolin vials with proper mixing. A 340 µl volume of kaolinized blood was added to disposable TEG cups that had pre-added 20 µl CaCl₂ (0.2 M) to initiate the coagulation cascade. Data were acquired in accordance with the manufacturer’s instructions until the maximum amplitude was achieved or 60 min had lapsed. The TEG parameters, such as R (reaction time), K (kinetics), α angle (rate of clot formation, i.e., ‘thrombin burst’) and MA (maximum amplitude) were recorded and analyzed.

### Mouse tail-bleeding assay

Age-matched Swiss albino mice (10–12 weeks old) weighing 18–20 g were intraperitoneally administered with either longdaysin (5 mg/kg) or the vehicle. After 30 min, mice were intraperitoneally anesthetized with a ketamine/xylazine cocktail. The distal 3-mm segment of the tail was amputated with a fresh surgical blade. The tail was immediately submerged in PBS (pre-warmed at 37 °C), and bleeding was monitored. ‘Bleeding time’ was recorded as the time required until the cessation of bleeding for ≥10 s. The experiment was terminated in case bleeding continued until 20 min after injury^37^.

### MTT assay for platelet viability

Mitochondrial metabolic activity in platelets was evaluated using the MTT assay as described previously^88–90^. Briefly, 100 µl aliquots of washed human platelets (2 × 10⁸ cells/ml) were dispensed into wells of 96-well flat-bottom microplate. Platelets were treated either with longdaysin or vehicle, followed by stimulation with thrombin as described. MTT (10 μl) was added to each well to the final concentration of 0.5 mg/ml and incubated for 3 h in dark at 37 °C. MTT is reduced by the mitochondrial enzymes of metabolically active cells into a purple insoluble product. The formazan crystals generated were thoroughly dissolved in 100 μl DMSO, and the absorbance was measured at 570 nm with a multimodal microplate reader (BioTek, model Synergy H1). Relative metabolic activity was calculated as a percentage of absorbance relative to the untreated control group. Resting (untreated) platelet population was assumed as 100% viable, whereas Triton X-100 (0.1%)-treated samples were served to have 2–3% viability while comparing negative control, buffer B without cells (0%).

### Statistical methods

Statistical analyses were conducted utilizing Prism 8.4.0 software (GraphPad). Multiple *t*-tests and repeated measures one-way analysis of variance (RM one-way ANOVA) or two-way ANOVA with Sidak’s multiple comparison test were employed for assessment. Statistical significance was established at *p* < 0.05. Data are expressed as mean ± standard error of the mean (SEM) derived from a minimum of three independent experiments sourced from distinct blood donors.

### Ethics statement

Fresh venous blood from healthy volunteers was collected under informed consent strictly in accordance with the recommendations and as approved by the Ethical Committee of the Institute of Medical Sciences, Banaras Hindu University. The study methodologies conformed to the standards set by the Declaration of Helsinki. The animal experiments were approved by the Central Animal Ethical Committee of the Institute of Medical Sciences, Banaras Hindu University (approval no. Dean/2017/CAEC/83). All procedures involving animals were conducted in strict accordance with relevant ethical guidelines. Efforts were made to minimize both the number of animals used and any potential suffering. All research involving human participants was conducted in compliance with applicable ethical standards and regulatory requirements.

### Reporting summary

Further information on research design is available in the Nature Portfolio Reporting Summary linked to this article.

## Supplementary information

Supplementary Information

Description of Additional Supplementary files

Supplementary Video-1

Supplementary Video-2

Supplementary Data 1

Reporting Summary

## Data Availability

All relevant data that support the findings of this study are within the paper. The raw numerical values are provided in Supplementary Data 1 (Excel file), and the uncropped original western blots are presented in Supplementary Fig. 4.
