# Supplementary material for: Casein kinase I isoforms contribute to platelet activation and thrombogenesis via RIPK3–MLKL signaling
Source: Commun Biol. 2025 Sep 30;8:1384. doi: 10.1038/s42003-025-08868-1 (PMC12485074; doi:10.1038/s42003-025-08868-1)
Supplement: Supplementary file 1 — Supplementary Information [file 42003_2025_8868_MOESM1_ESM.pdf]

(SUPPLEMENTARY MATERIAL)

Casein kinase I isoforms contribute to platelet activation and thrombogenesis via RIPK3–MLKL signalling

Vipin Singh, Mohammad Ekhlak, Susheel N. Chaurasia, and Debabrata Dash*

Centre for Advanced Research on Platelet Signaling and Thrombosis Biology, Department of Biochemistry, Institute of Medical Sciences, Banaras Hindu University, Varanasi-221005, Uttar Pradesh, India

* Correspondence: Prof. Debabrata Dash, Center for Advanced Research on Platelet Signaling and Thrombosis Biology, Department of Biochemistry, Institute of Medical Sciences, Banaras Hindu University, Varanasi-221005, Uttar Pradesh, India. Email: ddash.biochem@gmail.com

Supplementary material includes Methods, two Figures and Videos MP4 file

1. **Supple Fig. 1**

- Thrombin-induced PAC-1 binding and P-selectin exposure in aspirin-treated platelets.
- TRAP-induced platelet aggregation to assess functional responses.
- Ristocetin-induced platelet agglutination
- Intracellular reactive oxygen species (ROS) level in thrombin-stimulated platelets.
- Effects of longdaysin and D4476 on unstimulated platelets.
- Platelet viability measured across varying doses of longdaysin

2. **Supple Fig. 2**

- Thrombin-induced fluorescent fibrinogen binding to murine platelets in *ex vivo* and *in vivo* models.

- Measurement of intracellular calcium in murine platelets following thrombin stimulation.
- Confocal microscopy images of platelet thrombi formation.
- Non-kaolinated TEG in recalcified blood and Prothrombin Time

3. **Flow cytometric gating strategies**
4. **Supplementary videos for intravital microscopy**
5. **Supplementary Fig. 4 Original Uncropped western blot data**

Murine platelet isolation

Murine platelets were prepared following previously established protocols. Briefly, mice were anesthetized using a cocktail of ketamine (100 mg/kg) and xylazine (10 mg/kg) administered intraperitoneally. Blood was collected from the retro-orbital vein using heparin-coated capillary tubes into a trisodium citrate solution (9:1 ratio). The collected blood was diluted with modified Tyrode's buffer (20 mM HEPES, 134 mM NaCl, 2.9 mM KCl, 1 mM MgCl2, 0.34 mM NaH2PO4, 12 mM NaHCO3, 5 mM glucose, and 0.35 g/dL BSA; pH 7.4) and centrifuged at 100 × g for 15 minutes at room temperature to isolate platelet-rich plasma (PRP). Next, PRP was supplemented with 1 mM PGE1 and centrifuged at 800 × g for 7 minutes to obtain platelet pellets. These pellets were washed with modified Tyrode's buffer containing 1 mM PGE1 and finally pellets were resuspended in the same buffer for further use.

Flow cytometric measurement of intracellular ROS generation

Washed human platelets were incubated with 2′,7′-dichlorodihydrofluorescein diacetate (H_2DCFDA, 10 μM) for 30 min at RT to assess intracellular ROS levels. Following incubation, ROS generation was induced by stimulating the platelets with thrombin (0.25 U/mL). Prior to stimulation, the platelets were pretreated either with vehicle or CK1 inhibitors. The resulting

fluorescence of dichlorofluorescein, indicative of ROS production, was recorded, and intracellular ROS levels were quantified as the percentage of cells positive for DCF fluorescence.

Supplementary. Fig. 1

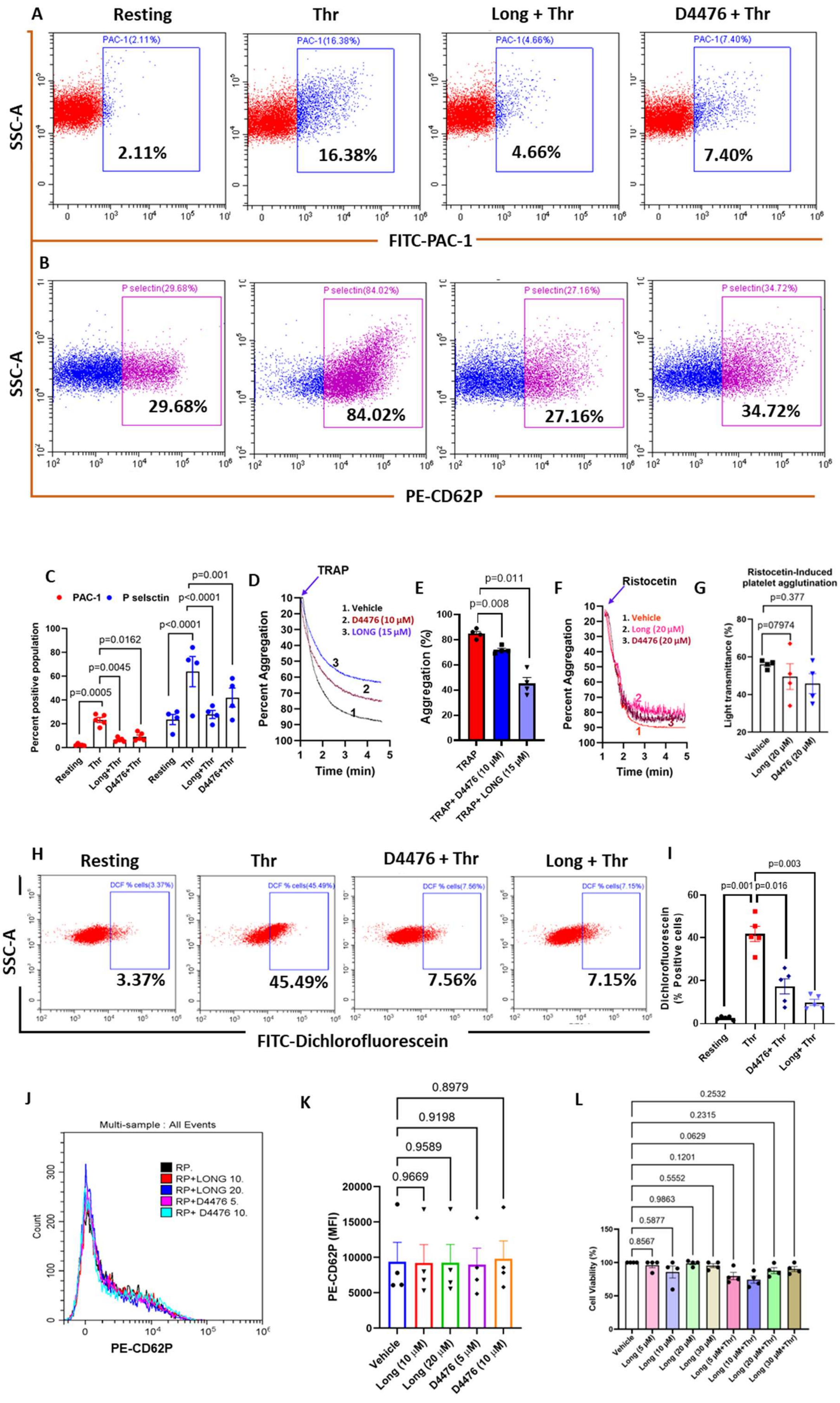

Supplementary Fig. 1. Effect of CK1 inhibition on function and viability of resting and stimulated platelets. A and B, binding of FITC-PAC-1 (A) and PE-CD62P (B) to human platelets pre-supplemented with acetylsalicylic acid (1 mM for 15 min) and exposed to different reagents as indicated. C, corresponding bar diagram representing percent positive populations for PAC-1 and CD62P binding in response to different reagents. D, TRAP (2 µM)-induced aggregation of human platelets with or without preincubation with longdaysin and D4476 as indicated. E, corresponding bar picture showing effect of CK1 inhibitors on TRAP-induced platelet aggregation. F, Tracing represents Ristocetin (1.25 mg/mL)-induced platelet agglutination. G, corresponding bar picture showing effect of CK1 inhibitors. H, ROS production in DCF-stained platelets exposed to different reagents as indicated. I, corresponding bar diagram demonstrating percent positive populations for DCFDA. J, histogram showing PE-CD62P binding to unstimulated platelets in the presence of vehicle, or varying concentrations of longdaysin or D4476 as indicated. K, corresponding bar picture showing mean fluorescent intensity of PE-CD62P binding to unstimulated platelets. L, bar diagram illustrates viability of human platelets preincubated with varying concentrations of longdaysin (5, 10, 20, 30 and µM) for 20 min at RT, followed by stimulation with thrombin. Data are presented as mean ± SEM and are representative of at least three different experiments.

Supplementary. Fig. 2

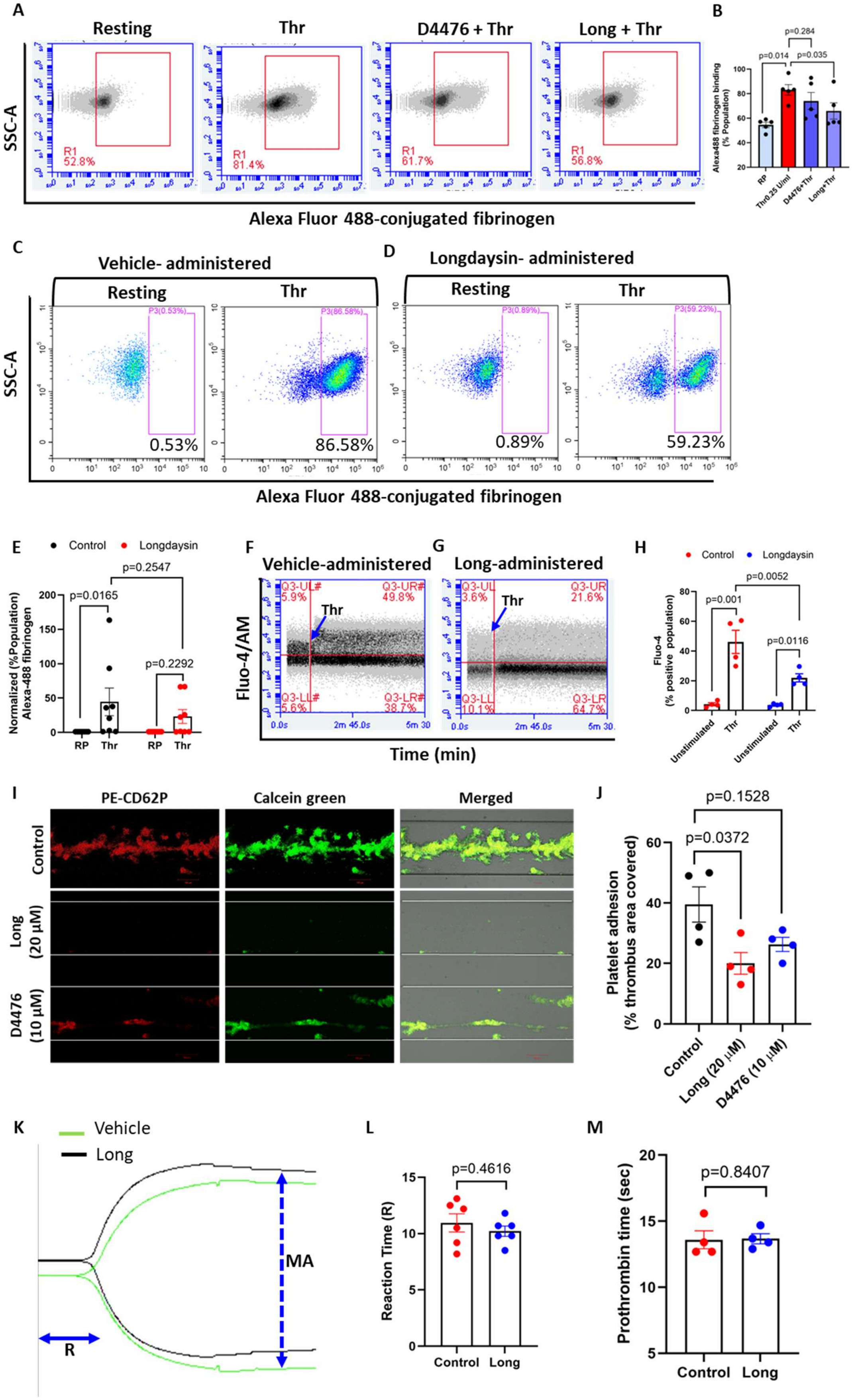

Supplementary. Fig. 2. CK1 Inhibition disrupts platelet activation but not extrinsic coagulation. A, thrombin-induced fluorescent fibrinogen binding to washed murine platelets pretreated *in vitro* with inhibitors of CK1, as indicated. B, corresponding bar diagram representing percent positive populations for Alexa fluor 488-fibrinogen recorded from the gated region (R1). C and D, thrombin-induced fluorescent fibrinogen binding to washed murine platelets isolated form mice pre-administered intravenously either with vehicle or longdaysin (5mg/kg). E, corresponding bar diagram representing the normalized percent positive populations for Alexa fluor 488-fibrinogen. F and G, thrombin-induced intracellular calcium flux against time in a population of Fluo-4-loaded murine platelets intravenously pre-administered either with vehicle or longdaysin. A prominent surge in Fluo-4 fluorescence upon thrombin addition (upper-right quadrant, Q3-UR) was observed in control cells (indicating calcium mobilization) whereas longdaysin-administered platelets exhibited a blunted calcium response. H, corresponding bar diagram representing mean of Fluo-4-positive events in platelet populations over a period of 4 min. I, confocal images exhibiting growth of platelet thrombi over collagen at 5 min following start of perfusion (at shear rate 1500 s^{-1}) in control (DMSO) as well as longdaysin (15 µM)-pretreated cells. J, corresponding bar diagram representing total surface area covered by platelet thrombi after 5 min perfusion over collagen matrix, studied in 4 representative fields (n = 4). K, thromboelastogram of non-kaolinated recalcified citrate human whole blood pre-incubated with either vehicle (green tracing) or with longdaysin (25 µM) (black tracing). L, corresponding bar diagram (n=6). M, representative bar picture denotes Prothrombin Time (sec) in vehicle vs longdaysin treated sample (n=4). Data are presented as mean ± SEM of at least 3 independent experiments. Statistical significance was determined using one-way ANOVA, two-way ANOVA and t-Test with appropriate post hoc tests.

Supplementary . Fig. 3

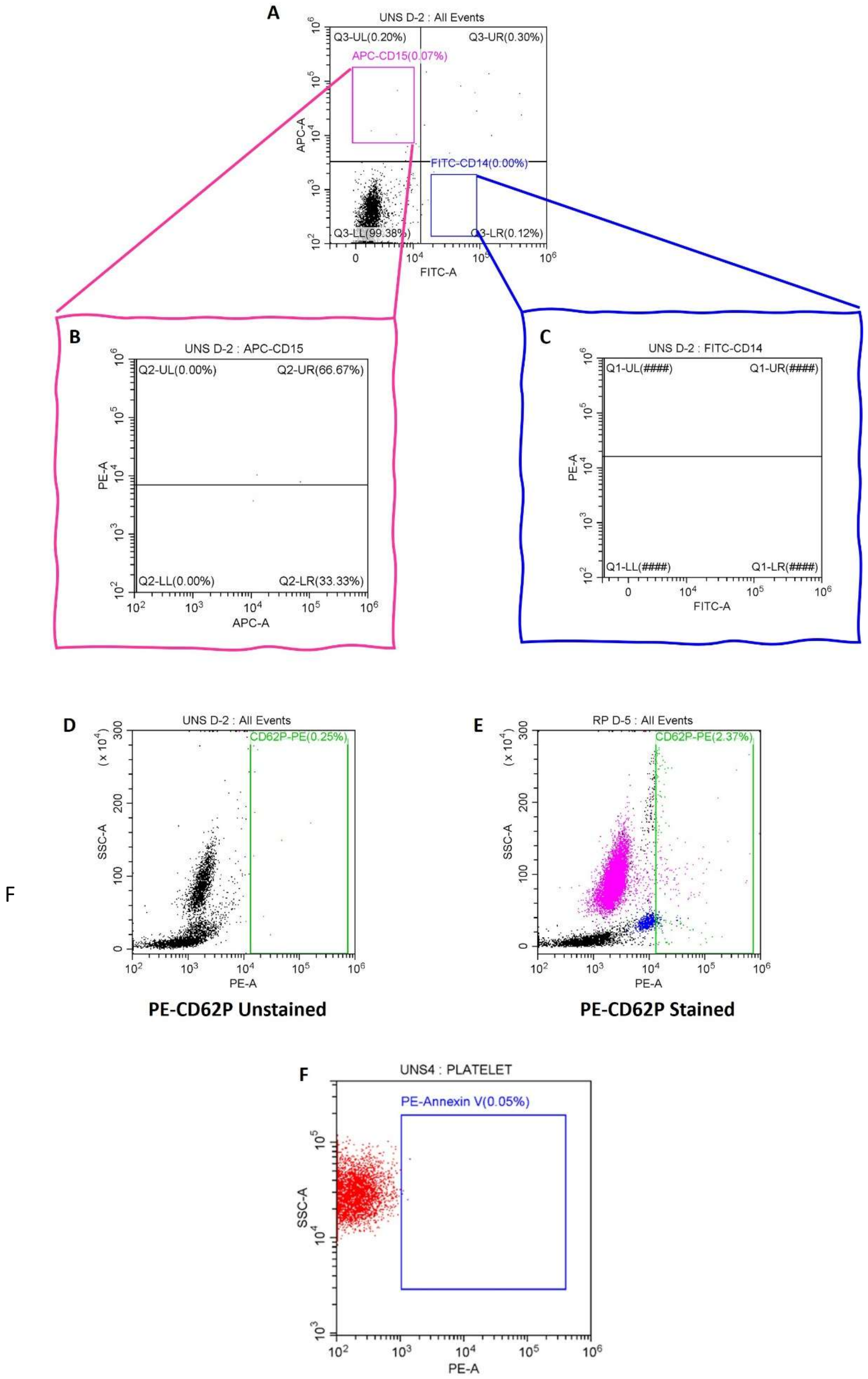

Supplementary Fig. 3, Flow cytometric gating strategies for analyzing platelet-leukocyte interactions. Monocyte, Neutrophil and platelets were gated using specific antibodies incubated with whole blood for 15 min either individually or as cocktail as described in the Methods section. A, quadrant dot plot represents unstained population for APC-CD15 (Neutrophil) and unstained FITC-CD14 (Monocyte) showing the initial gating to select all events of interest. B and C dot plot drawn with APC-CD15 and FITC-CD14 vs PE-CD62P to see the percent positive population for leucocyte and platelet interactions. D and E, region gated dot plots showing unstained vs-stained population for PE-CD62P. F, Region gated dot plot show annexin V negative populations in platelets unstained with the PE-annexin V.

4. **Supplementary videos for intravital microscopy**

Supplementary Video 1. Ferric chloride-induced mesenteric arteriolar thrombosis in mice pre-administered with vehicle (control). Platelets were fluorescently labelled with DyLight 488 anti-GPIbβ antibody (0.1 µg/g body weight).

Supplementary Video 2. Ferric chloride-induced mesenteric arteriolar thrombosis in mice pre-administered with longdaysin (5 mg/kg). Platelets were fluorescently labelled with DyLight 488 anti-GPIbβ antibody (0.1 µg/g body weight).

5. **Supplementary Fig. 4 Original Uncropped western blot data**

Supplementary Fig. 4

P-RIPK3 (46-62)

Uncropped blot for Figure 3, C

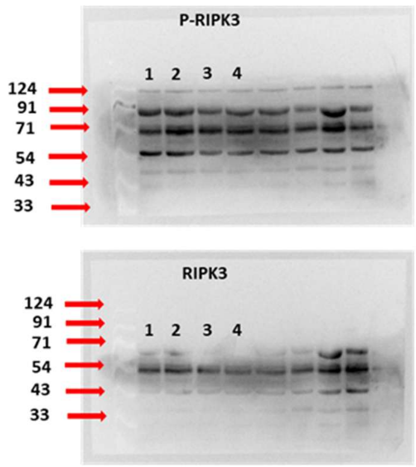

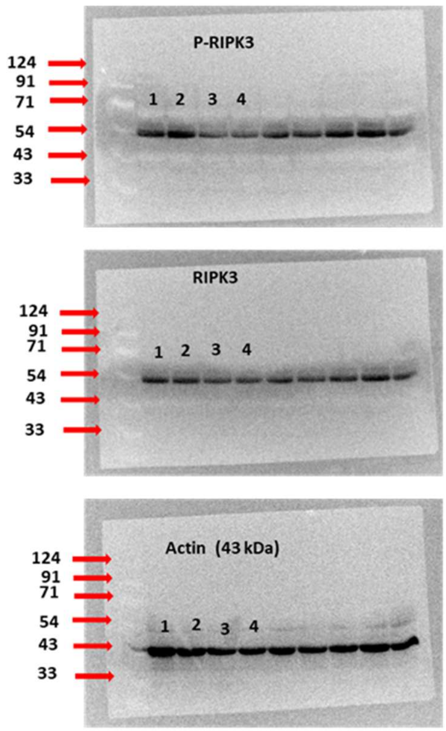

1. Resting
2. Thr
3. Gsk-872+ Thr
4. Long+ Thr

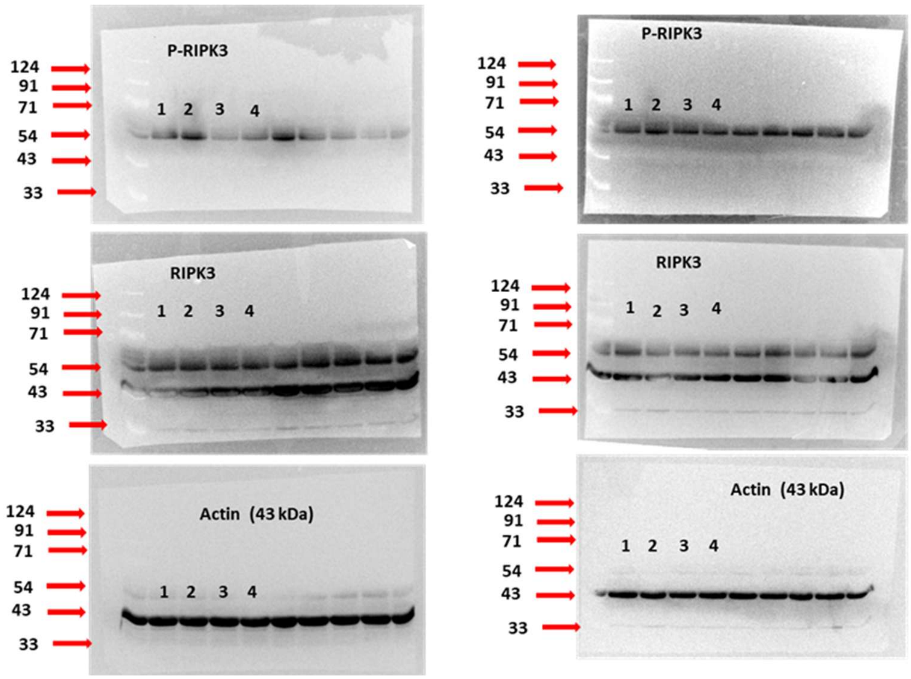

P-MLKL (54 kDa)

1. Resting
2. Thr
3. Gsk-872+ Thr
4. Long+ Thr

Uncropped blot for Figure 3, E

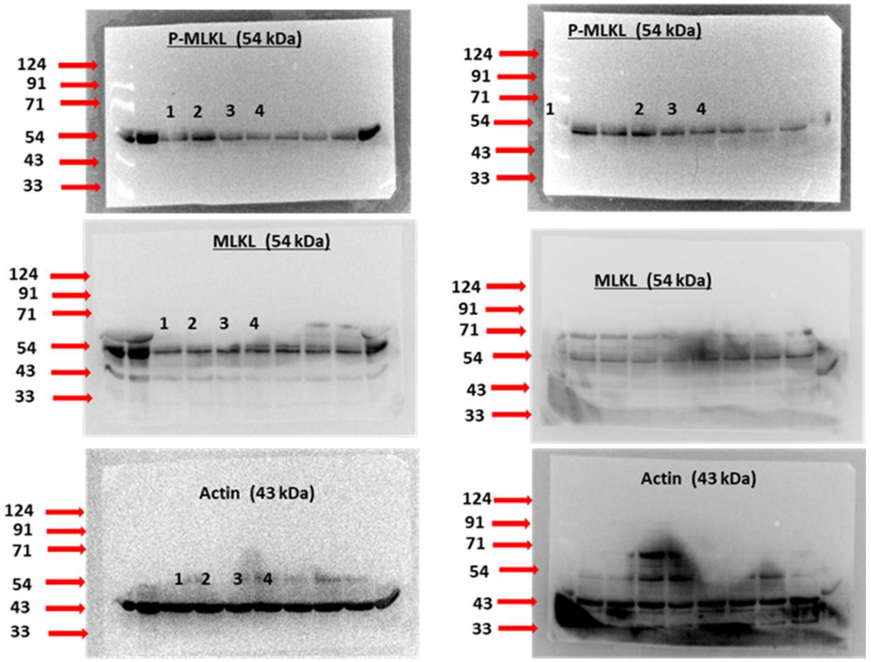

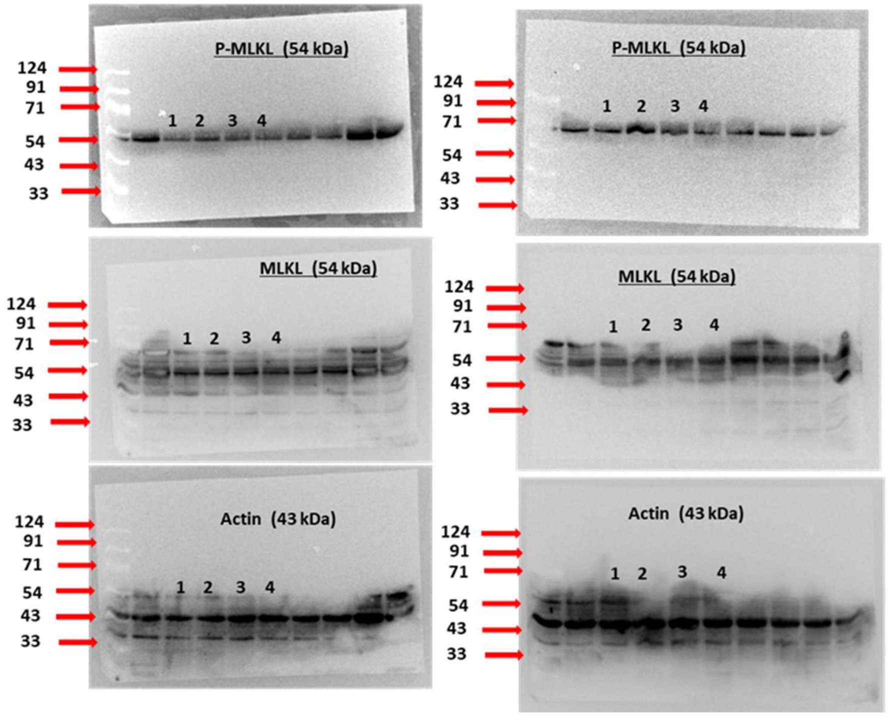

P-Src418 (59 kDa)

Uncropped blot for Figure 3, G

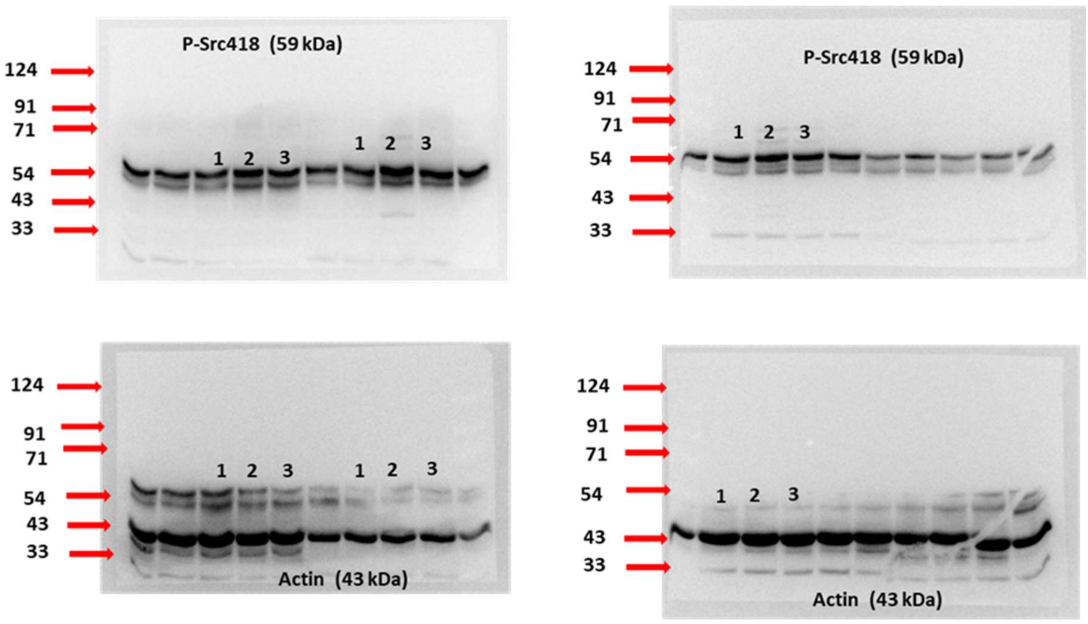

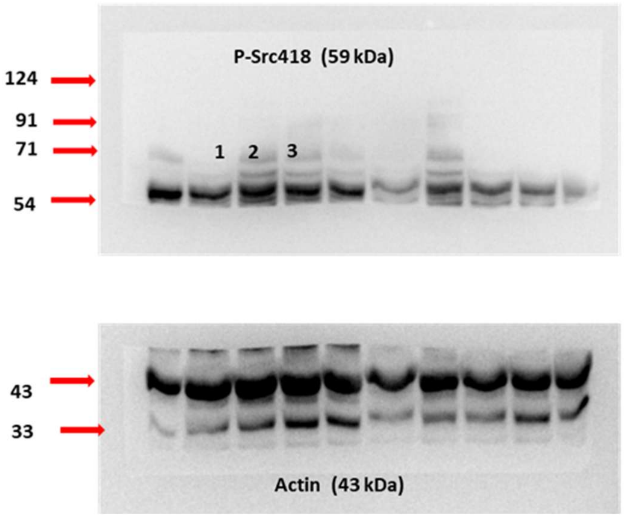

1. Resting
2. Thr
3. Long+ Thr

P-Akt (60 kDa)

1. Resting
2. Thrombin
3. LY 294002+ Thr
4. D4476+ Thr
5. Long+ Thr

Uncropped blot for Figure 3, I

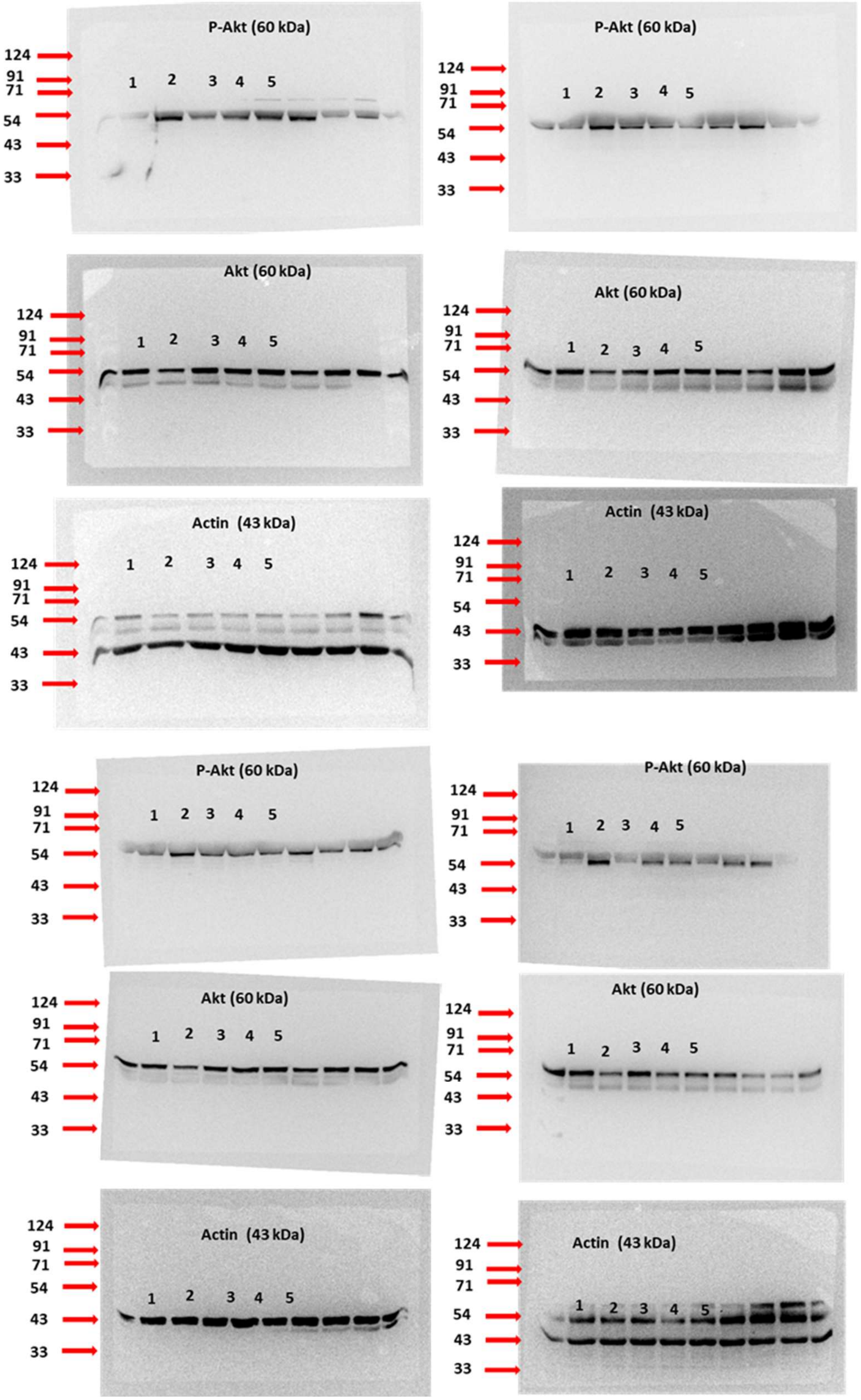

P-GSK3β (46)

1. Resting
2. Thrombin
3. LY 294002+ Thr
4. D4476+ Thr
5. Long+ Thr

Uncropped blot for Figure 3, K

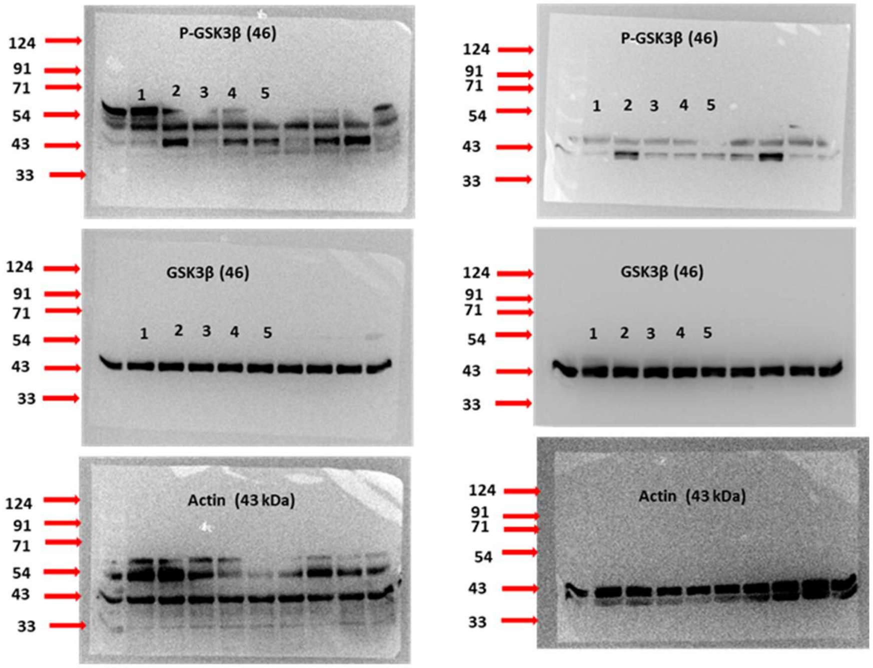

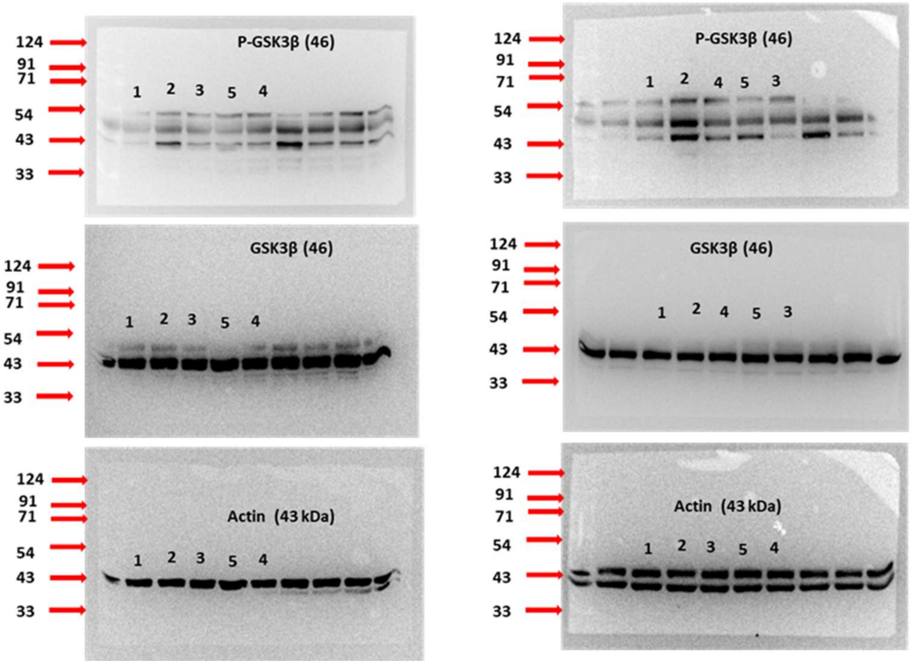

PY99 (60 to 120 kDa)

Uncropped blot for Figure 3, M

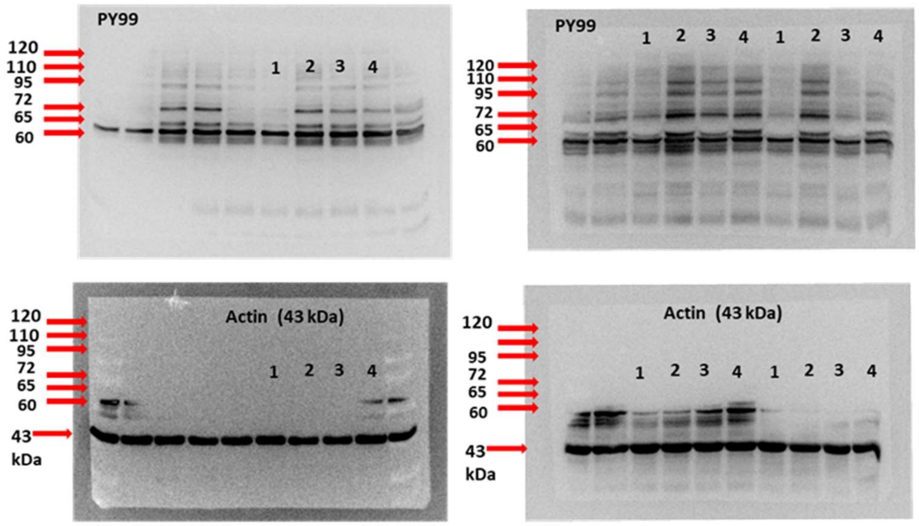

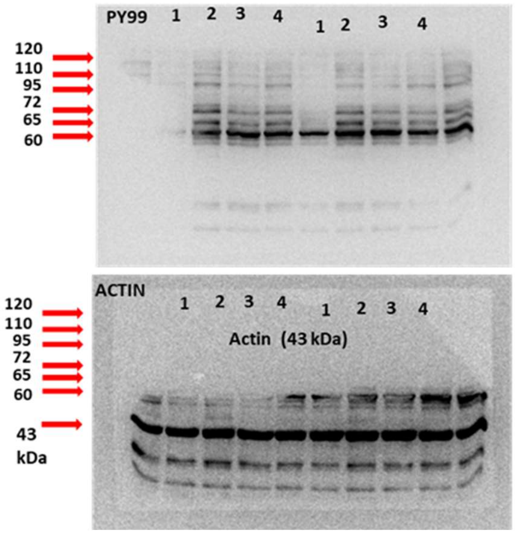

1. Resting
2. Thrombin
3. Long+ Thr
4. D4476+ Thr
