# Supplementary material for: Casein kinase I isoforms contribute to platelet activation and thrombogenesis via RIPK3–MLKL signaling
Source: Commun Biol. 2025 Sep 30;8:1384. doi: 10.1038/s42003-025-08868-1 (PMC12485074; doi:10.1038/s42003-025-08868-1)
Supplement: Supplementary file 2 — Description of Additional Supplementary files [file 42003_2025_8868_MOESM2_ESM.docx]

**Description of Additional Supplementary files**

**Supplementary Video- 1**

This video file presents live imaging of thrombus formation in mice administered with the vehicle control (DMSO)

**Supplementary Video- 2**

This video file shows live imaging of thrombus formation in mice administered with Longdaysin

**Supplementary Data 1 (Excel)**

This file contains all the numeric value for the statistical analysis performed in the manuscript, file attached as LaTeX file
